# Temporal transcriptomic analysis of scale drop disease virus in Asian seabass kidney cells reveals host immune and signaling changes

**DOI:** 10.3389/fimmu.2026.1819983

**Published:** 2026-06-09

**Authors:** Hong-Yi Xin, Lee Ching Pei Carmen, Lim Xin Ying, Mookkan Prabakaran

**Affiliations:** Temasek Life Sciences Laboratory, National University of Singapore, Singapore, Singapore

**Keywords:** Asian seabass kidney cells, host-virus interactions, scale drop disease virus (SDDV), transcriptomics, viral pathogenesis

## Abstract

Scale drop disease virus (SDDV), member of the genus *Megalocytivirus* within the family *Iridoviridae*, has emerged as a major viral threat in Asian seabass (ASB) (*Lates calcarifer)* aquaculture, causing severe mortality and significant economic impact. Progress in understanding SDDV pathogenesis remains limited by the lack of permissive *in vitro* models and comprehensive molecular investigations. In this study, we characterized host transcriptional responses to SDDV infection using a permissive ASB kidney (ASBK) cell system. Time-resolved RNA sequencing was performed at 8, 24 and 72 h post-infection to resolve the temporal dynamics of host gene regulation. Integrated analysis combining differential expression, protein-protein interaction (PPI) mapping, temporal clustering, and weighted gene co-expression network analysis (WGCNA) revealed distinct host responses across pathways and time. SDDV infection induced dynamic remodeling of immune signaling pathways, viral entry-associated processes, intracellular communication networks, programmed cell death pathways, and key signaling cascades. Early infection was characterized by rapid activation of innate immune sensing and signaling, whereas later stages were marked by immune modulation and cellular reprogramming. Together, this study establishes ASBK-1 cells as a robust platform for mechanistic studies of SDDV and elucidates the time-dependent host molecular networks during SDDV infection, thereby advancing our understanding of host-virus interactions in Asian seabass.

## 1 Introduction

Scale Drop Disease Virus (SDDV), a member of the genus *Megalocytivirus* within the family *Iridoviridae*, has emerged as a major viral threat to Asian seabass (ASB) (*Lates calcarifer*) aquaculture in Southeast Asia (SEA) (1). Although scale drop disease in ASB was observed as early as the 1990s, the viral etiological agent responsible for the disease was only isolated and molecularly characterized in 2015 (2). Since then, SDDV has been associated with recurrent disease outbreaks characterized by scale loss, fin erosion, lethargy, abnormal swimming behavior, and high mortality have been reported in Singapore, Malaysia, Thailand, and Indonesia (3, 4). In Singapore alone, SDDV-associated losses were estimated at SGD 31.9 million in 2022, highlighting the economic consequences of this disease (5). Beyond ASB, SDDV has also been reported in yellowfin seabream (*Acanthopagrus latus*) in China (1), indicating its potential to infect multiple fish species.

Despite its growing socio-economic impact, the molecular basis of SDDV infection, host-virus interactions, and disease progression remain poorly understood. Most studies to date have focused on epidemiology, diagnostics, vaccine development, and pathological characterization (6), with limited insight into host cellular signaling and immune regulation during infection. As a result, key questions remain regarding how SDDV interacts with host entry pathways, modulates immune signaling, and reprograms cellular processes to establish productive infection. Progress in molecular characterization has been further constrained by the limited availability of highly permissive *in vitro* systems. Moreover, traditional analytical approaches often lack sufficient resolution necessary to detect subtle yet biologically relevant transcriptional changes.

Advances in next-generation sequencing (NGS) technologies have enabled detailed characterization of host transcriptional responses during viral infection. Transcriptomic analyses facilitate the identification of differentially expressed genes (DEGs), elucidation of immune and signaling pathways, and discovery of host factors involved in viral replication and immune modulation. While such approaches have been widely applied to several fish viral pathogens (7–10), comprehensive and time-resolved transcriptomic studies of SDDV infection are limited.

**Figure 1:**
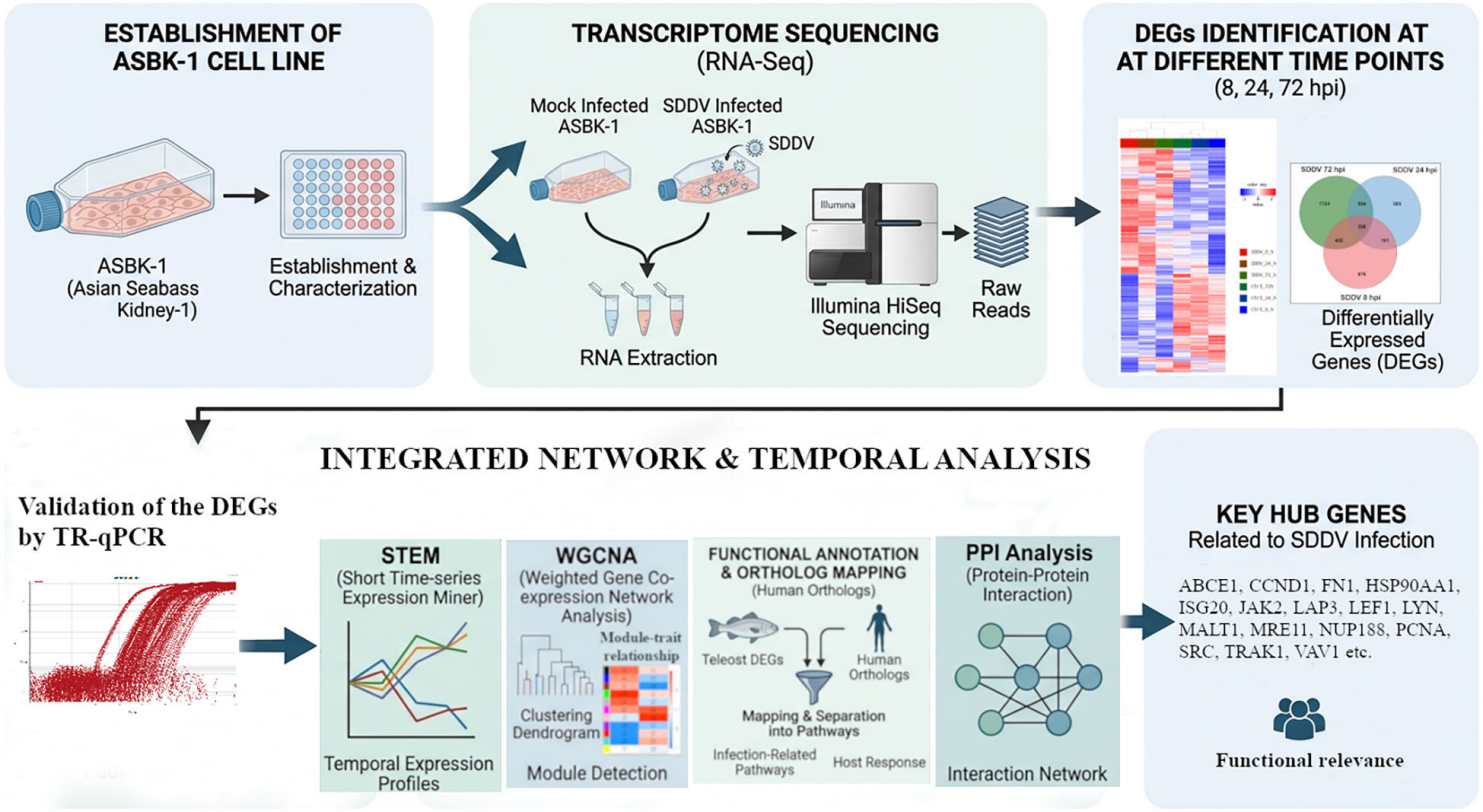
Integrated transcriptomic and temporal analysis workflow of the SDDV-infected Asian seabass kidney-1 (ASBK-1) cells.

In this study, we used a highly permissive ASB kidney (ASBK) cell model to investigate host transcriptional dynamics during SDDV infection. ASBK-1 cells were infected with an SDDV isolate, and RNA sequencing was performed at early, intermediate, and late stages of infection. Integrative analysis, including differential gene expression profiling, weighted gene co-expression network analysis (WGCNA), temporal clustering, and protein-protein interaction (PPI) network mapping, revealed dynamic and time-dependent host responses. The overall workflow is illustrated in **Figure 1**. Our findings demonstrate early activation of innate immune pathways, followed by progressive remodeling of key signaling and cellular processes during infection. Together these results provide new insights into SDDV-host interactions and establish ASBK-1 cells as a robust platform for mechanistic studies of viral pathogenesis.

## 2 Materials and methods

### 2.1 Establishment of ASBK cell line

Juvenile Asian seabass (*Lates calcarifer*) were euthanized by immersion in 500 mg/L tricaine methanesulfonate (MS-222; Sigma-Aldrich, USA). Kidney tissues were aseptically excised and transferred into Leibovitz’s L-15 medium (Gibco, USA) supplemented with antibiotic–antimycotic solution (Gibco, USA). The tissues were then minced and enzymatically dissociated in L-15 medium containing 0.25% trypsin at 30 °C for 30 min with gentle agitation. The cell suspension was filtered through a 100 µm mesh (Corning, USA), centrifuged at 300 × g for 10 min, and resuspended in growth medium consisting of L-15 supplemented with 20% fetal bovine serum (Gibco, USA), 1× antibiotic-antimycotic, and 100 µg ml^–1^ gentamicin sulfate (Sigma-Aldrich, USA). Cells were seeded into 25 cm² flasks and maintained at 28 °C, with 50% medium replacement every 3–4 days until 80–85% confluence. Subculturing was performed using 0.05% trypsin–EDTA at a 1:2 split ratio. Gentamicin was gradually withdrawn after 10 passages. Clonal populations were established by limiting dilution in 96-well plates, and the resulting cell line was continuously propagated and designated ASBK-1. The cells maintained a stable epithelial-like morphology during long-term culture.

### 2.2 Virus detection and isolation

Kidney samples were collected from diseased ASB (mean body weight ~150 g) obtained from an aquaculture farm in Singapore in 2023. Fish were euthanized using MS-222 as described above, and kidney tissues were aseptically excised for virus detection and isolation. Total nucleic acids were extracted using the PureLink Viral RNA/DNA Mini Kit (Invitrogen, USA) according to the manufacturer’s instructions. Viral infection was screened by PCR using primers targeting conserved regions of *Megalocytivirus* (11) and the SDDV capsid protein gene (**Table 1**). PCR amplicons were confirmed by Sanger sequencing as described previously (11). For virus isolation, kidney lysates were supplemented with 2x antibiotic–antimycotic solution and clarified by centrifugation at 4000 × g for 10 min. The resulting supernatant was filtered through a 0.45 μm membrane and inoculated onto ASBK-1 cell monolayers and incubated at 28 °C.

**Table 1:** Primers used in the study.

Primer name	Sequences
SDDV mCAP -F	ATGTCATCTATTGCAGGAGCTAATGTT
SDDV mCAP -R	TTACAAGATCGGAAATCCAAATGAACC
HPRT1-F	GGACTCATCTTGGACAGGACAG
HPRT1-R	ACTGTCATTGGGATGGAGCG
ABCE1-F	CGACTTCATCATGGCGACCT
ABCE1-R	ACAGACTCTGAGGCGTGTTA
ATR-F	GACCCCACGTCATGGTACAG
ATR-R	TCCATGGCGATCTCCCAAAC
CD151-F	AGGTGACCATGCAGCAGAAA
CD151-R	GCTGCCACAACACTTAAACTCC
FCHO1-F	CGTGCTCTACCACAATATGAAGC
FCHO1-R	TCAATGGCAGCCCTCTCACG
FN1a-F	TACCCTTGGTCAAGGTGAGC
FN1a-R	TGCCAGGAGATGGTTGTGAC
RUNX1-F	CGACGAGAACTACTCAGCGG
RUNX1-R	AGTGAAACTCTTCCCTCTTCCG
HDAC2-F	GTTTGAGAACCTGCGGATGC
HDAC2-R	CGGTGGCACGAATGGACAAG
SFRP1a-F	GCCAGCGAATTTGCTTTCAA
SFRP1a-R	GGCAATCCGCACCATTCTTC
HSP90AA1-F	AGGTCATCCGCAAGAACCTG
HSP90AA1-R	TCGTGGATGCCGAGCTTAAT
PHB-F	GCCACGCATCTTCACCAGTA
PHB-R	GGCATCAAACCGAGCCACTA
SMAD3-F	TGCATCACTATACCGAGGTCTCT
SMAD3-R	GTAGATGACGTGGGGCAGAC
HBEGF-F	TTACTCAGGCGAAAGGTGCG
HBEGF-R	CCCCGCTTGTGAAACCTGAG

Cells were monitored for cytopathic effects (CPE) under light microscopy (Olympus IX71). Infected cultures were harvested at 6 days post-inoculation and stored at −80 °C. Virus stocks were prepared by three freeze–thaw cycles, followed by clarification to collect viral supernatant. For viral propagation, sub-confluent ASBK-1 cells in 75 cm² flasks were infected at a multiplicity of infection (MOI) of 1 and incubated at 28 °C for 4–5 days. Viral titers were determined as the 50% tissue culture infectious dose (TCID_50_) in ASBK-1 cells (12). Briefly, 10-fold serial dilutions of viral samples were inoculated onto sub-confluent ASBK-1 monolayers in a 96-well culture plate and incubated at 28 °C for 5 days. CPE was recorded, and viral titers were calculated using the Reed and Muench method (13) and expressed as TCID_50_/mL.

### 2.3 Infection of ASBK-1 cells with SDDV and sample collection

Sub-confluent ASBK-1 monolayers grown in 6-well plates were infected with SDDV at an MOI of 0.25 and incubated for 1 h at 28 °C to allow viral adsorption. Mock-infected cells treated with culture medium alone served as controls for each collection time-point. After infection, cells were washed twice with PBS and maintained in fresh complete culture medium. Infected cultures were incubated at 28 °C and harvested 8, 24, 48, 72 and 96 hours post-infection (hpi). For viral titration, cells collected at each time point were subjected to freeze-thaw cycles, and viral titers were determined by TCID_50_ assay in ASBK-1 cells as described above. All experiments were performed in biological triplicates.

For transcriptomic analysis, a separate set of triplicate infections was established. Total RNA was extracted from both mock-infected and SDDV-infected ASBK-1 cells at 8, 24 and 72 hpi using the mirVana™ miRNA Isolation Kit (Thermo Fisher Scientific, Lithuania) according to the manufacturer’s instructions. RNA concentration and purity were measured using a NanoDrop 2000 spectrophotometer (Thermo Scientific, Waltham, MA, USA), and RNA integrity was assessed with an Agilent 2100 Bioanalyzer (Agilent Technologies, Santa Clara, CA, USA). Only samples with RNA integrity numbers (RIN) >8 were used for library preparation. Complementary DNA (cDNA) libraries were prepared using the TruSeq Stranded mRNA Library Prep Gold Kit (Illumina, San Diego, CA, USA). Paired-end 150 bp sequencing (PE150) was performed on the Illumina NovaSeq 6000 platform (Illumina Inc., USA). Library preparation and sequencing were carried out by Macrogen, Inc. (Seoul, Republic of Korea).

### 2.4 Differential gene expression analysis

Raw sequencing reads were processed using Trimmomatic for quality control. Adapter sequences were removed, and bases with Phred quality scores below Q30 were trimmed. A four-base sliding window was applied and reads with an average quality score below 15 or shorter than 36 bp were excluded. High-quality reads were aligned to the Asian seabass reference genome (TLL_Latcal_v3, GCF_001640805.2) using HISAT2 with default parameters. Library size normalization was performed using the trimmed mean of M-values (TMM) method implemented in the edgeR package. Differential expression analysis was conducted using the exactTest function in edgeR. Genes with an absolute fold change (FC) ≥ 2 and a false discovery rate (FDR) ≤ 0.05 were considered significantly differentially expressed. Functional annotation was performed using the DAVID platform. Pathway classification and enrichment analysis were carried out through the Reactome database (https://reactome.org/) and Gene Ontology (GO) (https://geneontology.org/). To facilitate pathway mapping, human orthologs were identified via DAVID, Ensembl and OrthoDB.

### 2.5 Temporal expression profiling

To characterize temporal expression patterns during the SDDV infection, DEGs identified at 8 hpi (immediate early), 24 hpi (early), and 72 hpi (late) were clustered by the Short Time-series Expression Miner (STEM) software (14). The parameters were set as follows: 1) Maximum Unit Change in Model Profiles between Time Points is 2, 2) Maximum number of model profiles is 50 (similar profiles will be merged) 3) Minimum absolute expression change of DEGs is no less than 1.0. The profiles with a *p*-value ≤0.05 were considered as significant. DEGs within each profile were further subjected to GO and Reactome pathway enrichment analysis.

### 2.6 Weighted gene co-expression network analysis

Weighted gene co-expression network analysis was performed using the WGCNA package in R to identify biologically relevant co-expression modules. A total of 5,076 genes were initially assessed. Genes with variance below the 20th percentile were removed, resulting in 4,056 genes for network construction. A signed co-expression network was constructed. The soft-thresholding power was determined based on the scale-free topology criterion. The adjacency matrix was transformed into a topological overlap matrix (TOM) to assess network interconnectedness. Genes were hierarchically clustered based on TOM dissimilarity, and modules were identified using a minimum module size of 30 genes. To identify modules driving host-pathogen interaction, we constructed a clinical trait matrix using a binary categorical variable for infection status (mock vs. SDDV) and a continuous variable representing time (8, 24, 72 hpi). Module-trait relationships were assessed using Pearson’s correlation. Gene significance (GS) and module membership (MM) were calculated. Modules showing an absolute correlation coefficient >0.8 (*p* < 0.05) with infection status were designated as hub modules. Hub genes were defined as those with MM ≥ 0.8 and GS ≥ 0.5. Genes within hub modules were subjected to GO and Reactome.

### 2.7 Protein–protein interaction network and hub gene identification

Protein–protein interaction (PPI) networks were generated using the STRING database (https://string-db.org/) with a confidence threshold of ≥0.7. Networks were visualized in Cytoscape (v3.10.3). Hub genes were identified through the cytoHubba plugin by integrating results from 12 topological algorithms. For STEM-derived clusters, the top 10 genes ranked by each algorithm were selected. For functional/pathway-specific gene sets, the top 3 genes identified by each algorithm were retained.

### 2.8 Validation of DEGs by RT-qPCR

To validate the technical and biological reliability of the NGS data, we selected 12 candidate DEGs, including six significantly upregulated and six significantly downregulated genes, for quantitative PCR (qPCR) validation. The objective was to assess whether the transcriptional trends observed in the Illumina sequencing data were consistent when measured using an independent quantification platform. Primers were designed with Primer-BLAST (**Table 1**), and HPRT1 was used as the endogenous reference gene. Each 10 μL reaction contained 0.5 μL cDNA template, 0.5 μL each of forward and reverse primers, 5 μL of PowerUp™ SYBR™ 2 x Master Mix (Thermo Fisher Scientific, USA), and 3.5 μL nuclease-free water. The qPCR was performed using QuantStudio 5 Real-Time PCR System (Applied Biosystems). Thermal cycling conditions included uracil-DNA glycosylase (UDG) activation at 50 °C for 2 min, initial denaturation at 95 °C for 10 min, followed by 40 cycles of denaturation at 95 °C for 15 s, annealing at 60 °C for 15 s, and extension at 72 °C for 30 s. All reactions were performed in triplicates with three independent biological replicates. Relative gene expression was calculated using the 2^- ΔΔCt^ method.

### 2.9 Statistical analysis

Statistical approaches applied to transcriptome analyses (WGCNA, STEM, and PPI) are described in the respective sections above. All other statistical analyses were performed using GraphPad Prism (version 8.0.2). Differences between groups in RT-qPCR were assessed using the Wilcoxon rank-sum test. In the viral growth kinetic profile, differences across infection time points were assessed using one-way ANOVA followed by Tukey’s multiple comparison test. A *p-*value < 0.05 was considered statistically significant.

## 3 Results

### 3.1 Virus isolation and replication kinetics in ASBK-1 cells

SDDV was successfully isolated from the kidney tissues of diseased Asian seabass and propagated in the established ASBK-1 cell line. Following infection, characteristic CPE, including cell rounding and detachment, were observed as early as 24-48 hpi. By 72–96 hpi, extensive cell detachment and monolayer disruption were observed (**Supplementary Figure 1**).

**Figure 2:**
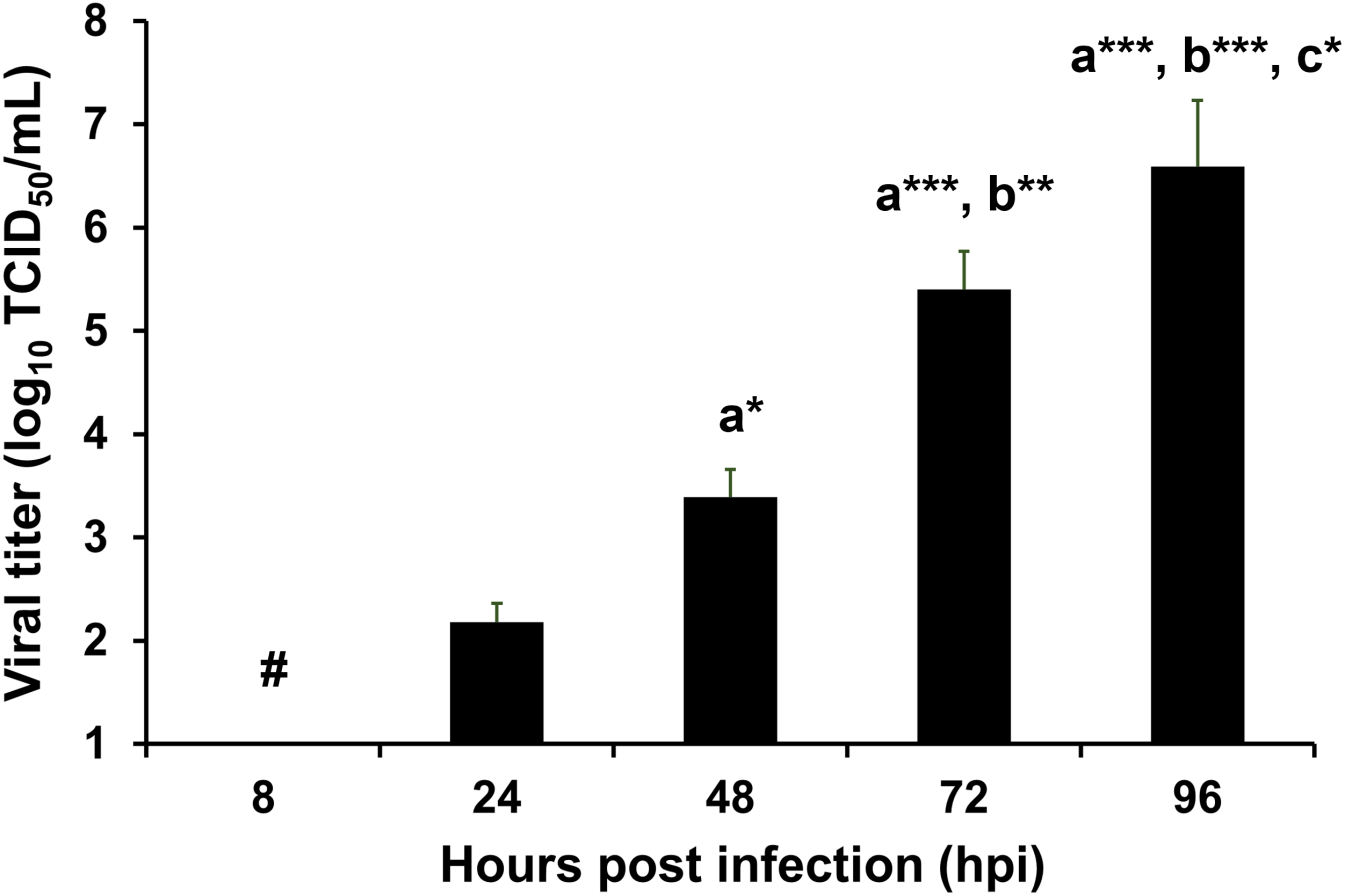
Growth kinetics of SDDV in ASBK-1 cells. Viral titers were determined at 8, 24, 48, 72, and 96 hpi and expressed as log_10_ TCID_50_/mL. Each time point represents the mean ± SD of three independent experiments. Statistical analysis showed that viral titers differed significantly across time points (24, 48 and 96 hpi) (*p* < 0.05). The symbol (#) indicates viral titers below the detection limit (<10^1^ TCID_50_/mL). Statistical analysis was performed using one-way ANOVA followed by Tukey’s multiple comparison test. Different letters indicate statistically significant differences (a, compared with 24 hpi; b, compared with 48 hpi; c, compared with 72 hpi) (*p < 0.05; **p < 0.01; ***p < 0.001).

To evaluate viral replication kinetics, viral titers were quantified using TCID_50_/mL. As shown in **Figure 2**, viral titers were below the detection limit at 8 hpi. Viral titers increased significantly over the observation period (24, 48 and 96 hpi). Detectable viral replication was observed at 24 hpi, followed by a progressive increase. Viral titers reached > 10^5^ TCID_50_/mL at 72 hpi and 10^6.5^ TCID_50_/mL at 96 hpi, indicating efficient viral replication in ASBK cells even at 0.25 MOI.

### 3.2 Transcriptomic analysis of SDDV-infected ASBK-1 cells

**Figure 3:**
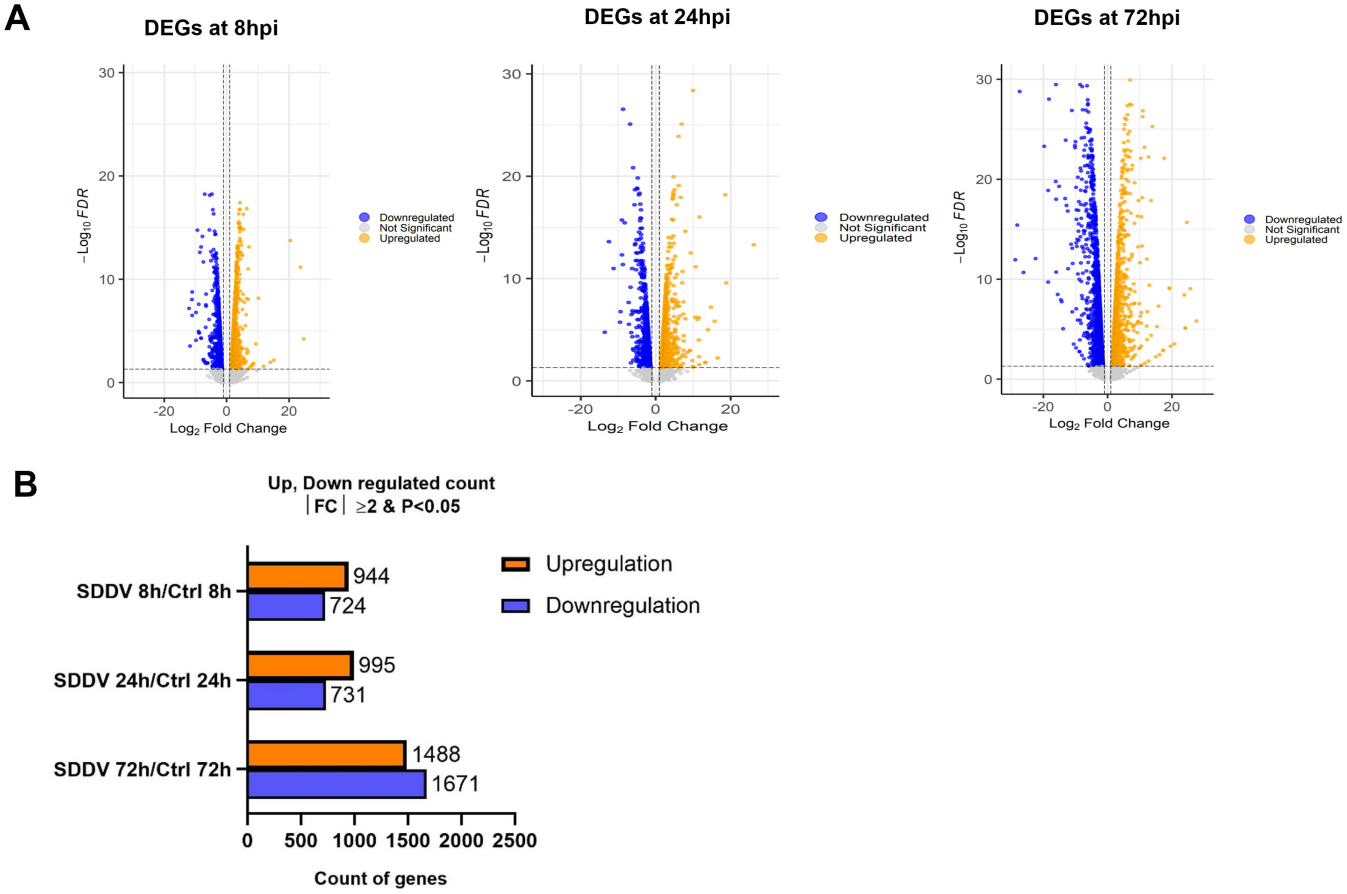
**(A)** Volcano plots show significantly upregulated and downregulated genes at each time point. The x-axis indicates log_2_ fold change (SDDV-infected vs. control) and y-axis represents –log_10_ (FDR). Each dot corresponds to an individual gene, orange dots indicate significantly upregulated genes, blue dots indicate significantly downregulated genes, and gray dots represent non-significant genes. **(B)** Temporal dynamics of DEGs during SDDV infection. Bar chart showing significantly upregulated (orange) and downregulated (blue) genes at 8, 24, and 72 hpi (FC ≥ 2, FDR < 0.05).

Differential gene expression (DGE) analysis was performed by comparing SDDV-infected and mock-infected ASBK-1 cells at 8, 24 and 72 hpi. Two-way hierarchical clustering demonstrated clear segregation between infected and mock-infected control groups at all time points (**Supplementary Figure 2**). Volcano plots illustrated the global distribution of DEGs (**Figure 3A**). At 8 hpi, 1,668 DEGs were identified (FDR <0.05, FC≥2), including 944 upregulated and 724 downregulated genes (**Figure 3B**; **Supplementary Table 1**). At 24 hpi, 1,726 DEGs were detected (FDR < 0.05, FC≥2), of which 995 were upregulated and 731 were downregulated (**Figure 3B**; **Supplementary Table 1**). The number of DEGs increased substantially at 72 hpi, with 3,159 genes differentially expressed (FDR < 0.05, FC≥2), comprising 1,488 upregulated and 1,671 downregulated genes (**Figure 3B**; **Supplementary Table 1**), indicating progressive transcriptional remodeling during the course of infection.

### 3.3 Weighted gene co-expression network analysis

WGCNA was employed to characterize the coordinated transcriptional architecture of the host response during SDDV infection. By partitioning the transcriptome into co-expression modules, this approach mitigates the ‘enrichment dilution’ effect often caused by high background noise within the total DEG pool. This strategy enabled the identification of highly specific infection-associated pathways that might otherwise remain obscured in a global, non-targeted analysis.

**Figure 4:**
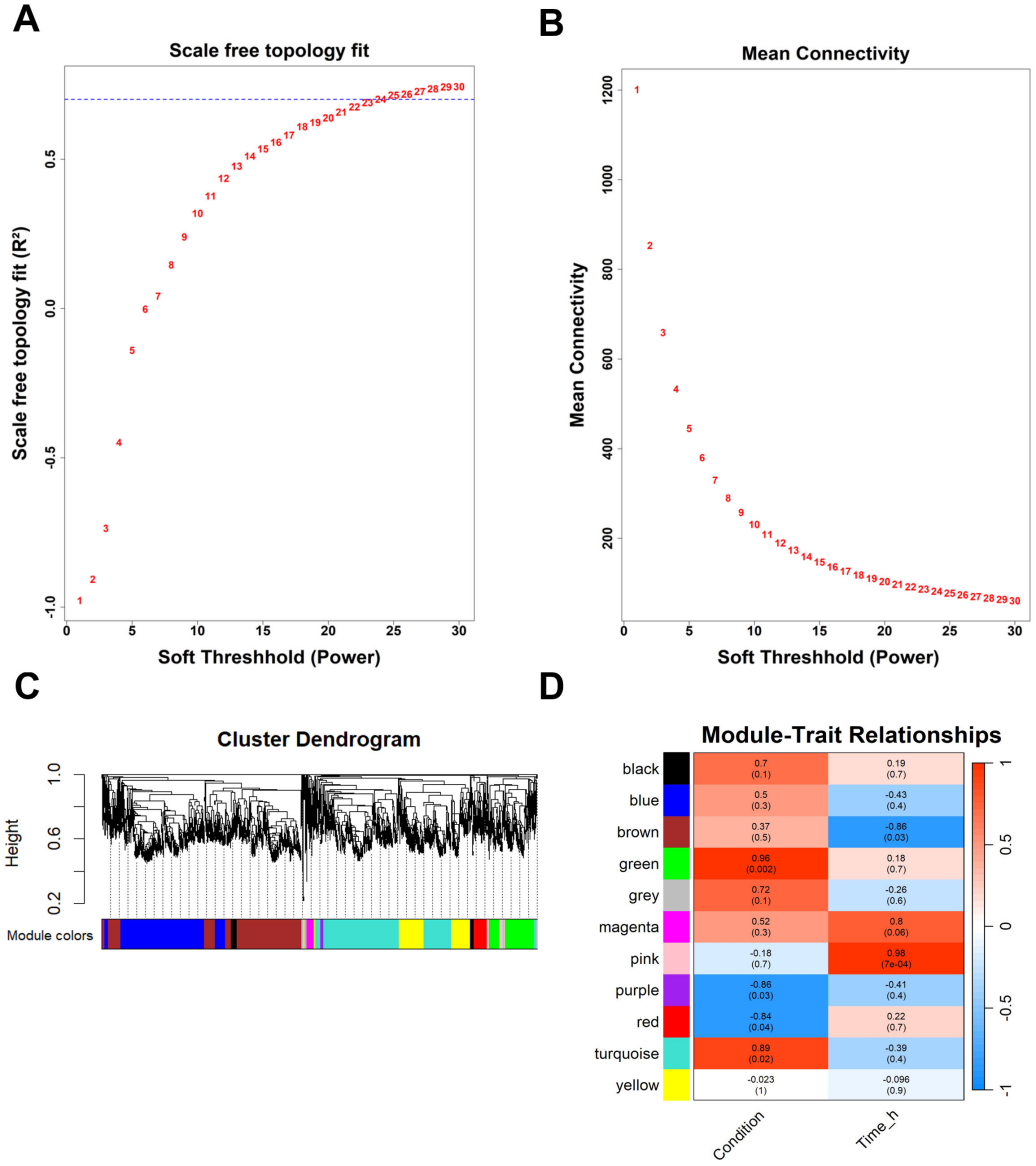
Weighted gene co-expression network analysis. **(A)** Scale-free topology model fit index (R^2^) as a function of soft-thresholding power (β). The horizontal dashed line indicates the threshold for scale-free topology fit (R^2^ = 0.7). **(B)** Mean network connectivity (degree, y-axis) as a function of soft-thresholding power (x-axis). Increasing β improves scale-free topology fit but reduces network connectivity. A power of 25 was selected as it achieved R^2^>0.7 while preserving adequate mean connectivity for downstream module detection. **(C)** The cluster dendrogram of genes. Each branch in the figure represents one gene, and each module was assigned a color in hierarchical clustering dendrogram. **(D)** Heatmap of module-infection time correlation: The value in the brackets indicates the p value and the value above the brackets represents the Pearson correlation coefficient (R value). The color of the bar represents the level of correlation.

To identify coordinated transcriptional changes during SDDV infection, we performed WGCNA. The soft-thresholding power (β) was selected based on scale-free topology fit (**Figure 4A**) and mean connectivity (**Figure 4B**). A power of 25 was selected to achieve a scale-free topology fit index (R²) > 0.7 while preserving sufficient network connectivity for reliable module detection. Hierarchical clustering combined with the Dynamic Tree Cut algorithm partitioned the transcriptome into 11 discrete co-expression modules, with module sizes ranging from 43 to 1027 genes. **Figure 4C** shows the cluster dendrogram used to identify co-expression modules, which were defined using the Dynamic Tree Cut algorithm with a minimum module size of 30 genes. Correlation analysis between module eigengenes and experimental traits identified several infection-responsive modules (**Figure 4D**). The green module (r = 0.96, *p* = 0.002) and the turquoise (r = 0.89, *p* = 0.02) showed a strong positive correlation with SDDV infection status. In contrast, purple (r = −0.86, p = 0.03), and red (r = −0.84, *p* = 0.04) modules were negatively correlated with infection. Two modules were primarily associated with time. The pink module showed a strong positive correlation (r = 0.98, *p* = 7 × 10^-4^), whereas the brown module exhibited a significant negative correlation with time (r = −0.86, *p* = 0.03), indicating time-dependent transcriptional regulation. However, eigengene expression patterns of these time-associated modules did not clearly distinguish infected from control samples. This suggests that temporal expression changes were not exclusively driven by SDDV infection progression over time.

Intramodular hub genes were identified within modules significantly associated with SDDV infection (**Supplementary Table 2**). Functional enrichment analysis (**Supplementary Figure 3**; **Supplementary Table 3**) showed that genes in the green module were enriched in TP53 signaling and cell cycle arrest pathways. The red module was enriched for host immune response pathways, while genes in the turquoise module were associated with immune response, cell cycle processes, cell adhesion, and cell growth.

### 3.4 Temporal expression profiling and functional annotation of DEGs

**Figure 5:**
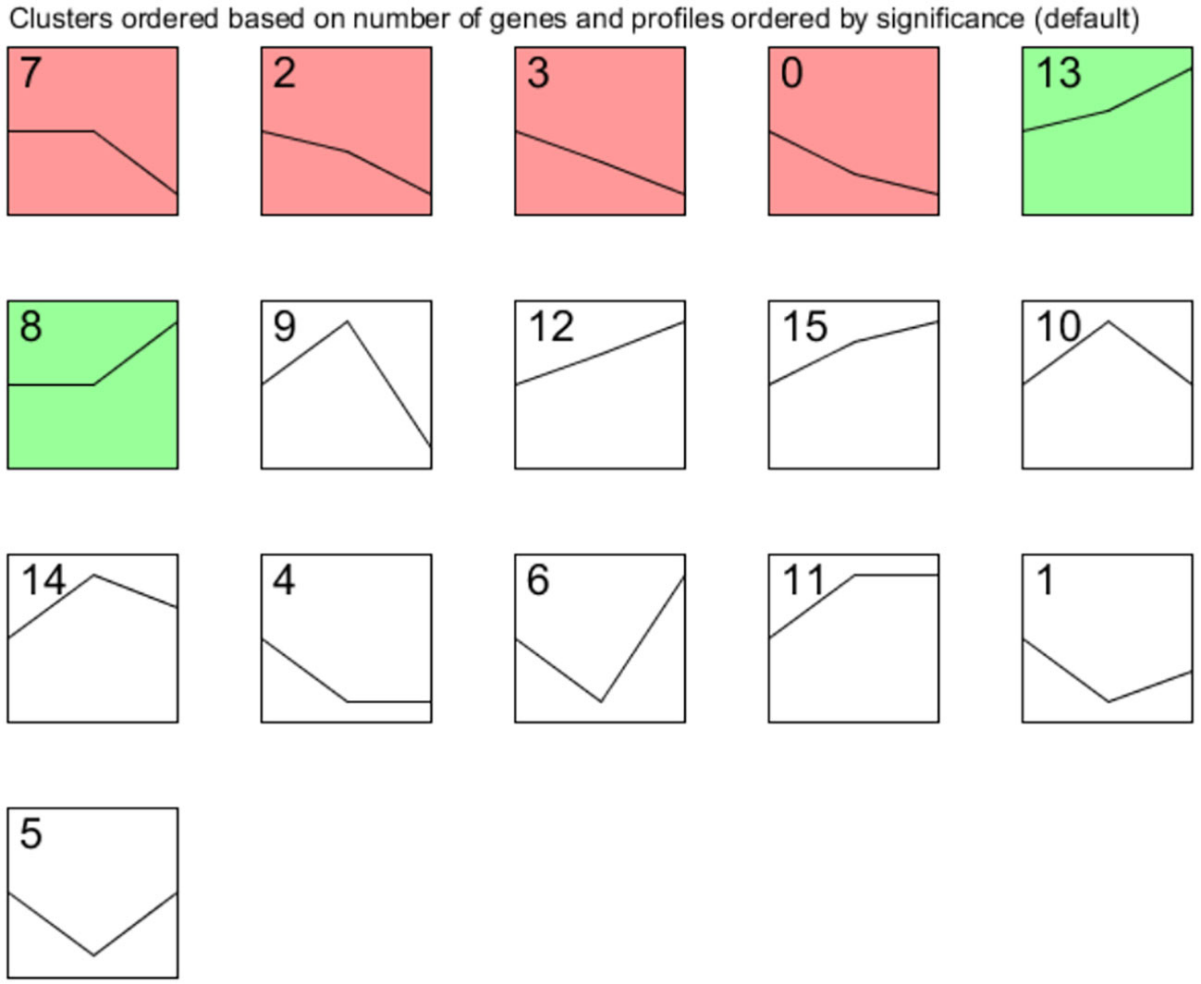
Trend analysis for DEGs at 8, 24 and 72 hpi. The 16 expression profiles obtained comparing DEGs at 8, 24 and 72 hpi.

**Figure 6:**
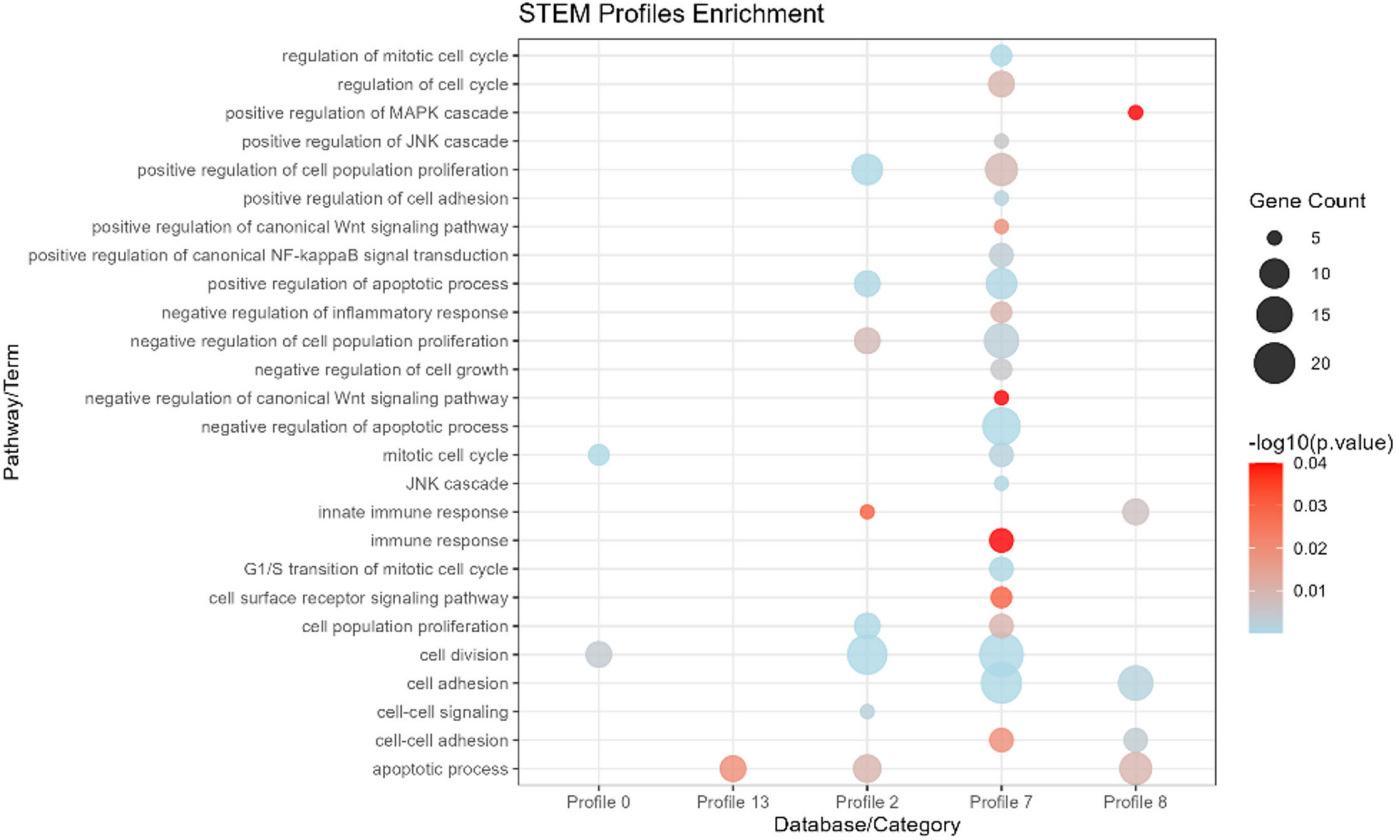
Enrichment analysis of the STEM profiles. Each bubble corresponds to an enriched biological term. Bubble size reflects the number of genes assigned to that term, while bubble color represents statistical significance, shown as –log10(*p*-value).

Temporal trend analysis was performed using DEGs at 8 hpi (immediate early), 24 hpi (early), and 72 hpi (late). Short Time-series Expression Miner (STEM) analysis identified 16 distinct temporal expression profiles (**Figure 5**), of which six (0, 2, 3, 7, 8 and 13) were significant (p< 0.05). DEGs in each significant profile are provided in **Supplementary Table 4**. Profiles 8 and 13 exhibited an uptrend regulation and were grouped into the green cluster. GO enrichment analysis of these upregulated profiles revealed enrichment (raw *p* < 0.05) in pathways related to innate immune response, apoptotic processes, cell adhesion, and positive regulation of the MAPK cascade (**Supplementary Table 5**). In contrast, profiles 0, 2, 3 and 7 exhibited a downtrend regulation and were grouped into the red cluster. GO enrichment analysis of these downregulated profiles revealed significant enrichment (*p <*0.05) in immune response, cell population proliferation, apoptotic processes, cell adhesion, cell-cell signaling, inflammatory response, cell division, canonical Wnt signaling, JNK cascade regulation, TOR signaling, and NF-kB signaling pathways (**Figure 6**; **Supplementary Table 5**).

STEM analysis grouped DEGs with similar temporal expression patterns into distinct clusters, reflecting coordinated transcriptional programs. Genes within the same cluster are likely co-regulated and may participate in related biological pathways. PPI network analyses were conducted separately for genes within each temporal cluster, resulting in the identification of hub genes (**Supplementary Table 6**). The hub genes were further mapped to the segregated pathways to find the key hub genes (**Supplementary Figure 4**).

### 3.5 Identification of orthologs and segregation of DEGs

Because functional annotations are limited for ASB, human ortholog-based enrichment was applied. Orthologous relationships were retrieved using DAVID (Database for Annotation, Visualization, and Integrated Discovery), and only high-confidence orthologs (confidence score = 1) were retained. Gene names are presented using human ortholog nomenclature throughout, with corresponding ASB gene identifiers provided (**Supplementary Table 7**).

DEGs were mapped to several important pathogen infection-related biological pathways through the curated Reactome (https://reactome.org, accessed August 28, 2025) (15). These pathways include immune response, programmed cell death, cell-cell communication, regulation of cell cycle arrest, cell adhesion and endocytic trafficking, and infection-related signaling pathways including PI3K/AKT (**Supplementary Table 8**). The PPI and hub genes within each category were analyzed using Cytoscape with the cytoHubba plugin (**Supplementary Figures 5**-**S8**). These genes were subsequently integrated with hub genes identified by WGCNA and STEM, yielding the final set of total hub genes mapped to the respective biological pathways (**Supplementary Table 9**).

### 3.6 Dynamic immune signatures induced during SDDV infection

**Figure 7:**
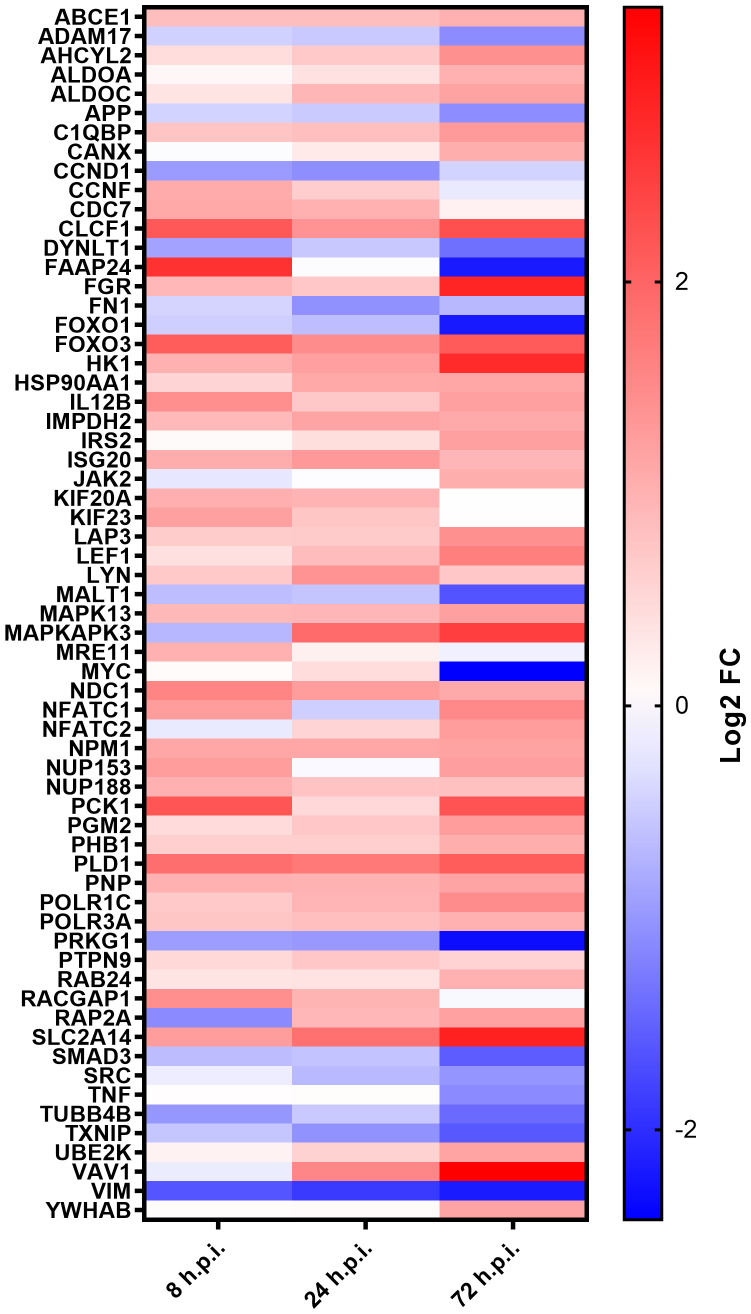
Heatmap of immune-related hub genes during SDDV infection.

Integrated WGCNA, STEM, and pathway analyses identified a total of 63 immune-related hub genes (**Figure 7**; **Supplementary Table 9**). In WGCNA, 28 immune-related hub genes were clustered within the turquoise module. STEM analysis indicated that 36 genes showed a progressive downregulation and 25 genes showed progressive upregulation across the SDDV infection time course.

In our dataset, several immune-related hub genes displayed either transient or sustained expression changes. At 72 hpi, ten hub genes were significantly downregulated, whereas twenty-two hub genes were significantly upregulated. Seven genes exhibited sustained upregulation across time points. In contrast, VIM was significantly downregulated at all examined time points. Notably, FAAP24 showed a biphasic expression pattern (upregulated at 8 hpi followed by downregulation at 24 and 72 hpi), whereas RAP2A showed an inverse temporal pattern, with early downregulation at 8 hpi and subsequent upregulation at 24 hpi, reaching significant upregulation by 72 hpi.

### 3.7 Dynamic regulation of DEGs associated with membrane trafficking and host response to infection

**Figure 8:**
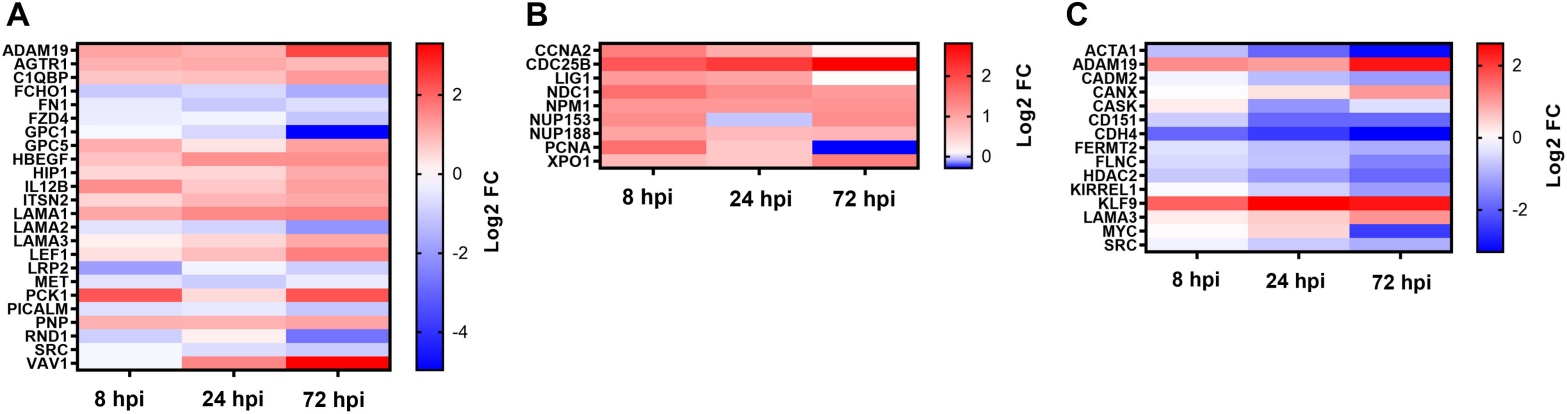
**(A)** Heat map of the hub genes in membrane trafficking and host response to infection. **(B)** Heat map of the hub genes involved in cell cycle arrest. **(C)** Heat map of the hub genes involved in cell-cell communication.

Among the DEGs associated with membrane trafficking and cell-surface regulation, 24 hub genes were identified that are involved in cell attachment, cell adhesion, and cell endocytosis (**Figure 8A**; **Supplementary Table 9**). Of these, 9 genes were assigned to the turquoise module in the WGCNA analysis and showed a strong positive correlation with SDDV infection (r = 0.89, *p* = 0.02). Temporal expression profiling revealed dynamic regulation patterns during infection. Seven genes were significantly upregulated at 8 hpi, whereas FCHO1 and LRP2 were significantly downregulated at this early time point. At 72 hpi, 6 genes were significantly downregulated, while 5 genes were significantly upregulated at this late stage. Notably, ADAM19 and LAMA1 were significantly upregulated throughout the entire course of SDDV infection. STEM trend analysis further demonstrated progressive temporal regulation: FCHO1, LAMA2 and GPC1 showed progressive downregulation over time, whereas VAV1, ADAM19, LEF1 and LAMA3 showed progressive upregulation. Especially, VAV1 displayed a biphasic response, with significant downregulation at 8 hpi followed by upregulation at 24 and 72 hpi.

### 3.8 Dynamic regulation of DEGs involved in cell cycle arrest

Cell cycle arrest plays a dual role in host–pathogen interactions. From the host perspective, arrest can limit viral spread by suppressing cell proliferation, activating DNA damage responses, and facilitating innate immune signaling. Conversely, many viruses induce or maintain cell cycle arrest to establish a replication-permissive intracellular environment. In our analysis, 9 hub genes were associated with cell cycle arrest (**Figure 8B**; **Supplementary Table 9**). Of these, 8 genes were clustered into turquoise module, whereas CCNA2 was assigned to the green module in the WGCNA analysis. All hub genes, except XPO1, were upregulated at 8 hpi. NDC1, NPM1 and CDC25B exhibited sustained upregulation throughout the course of SDDV infection. STEM analysis revealed progressive downregulation of CCNA2, PCNA and LIG1, whereas CDC25B and XPO1 showed progressively increasing expression trends.

### 3.9 Dynamic regulation of DEGs involved in cell–cell communication

A total of 15 hub genes were identified within the cell-cell communication network (**Figure 8C**; **Supplementary Table 9**). In WGCNA analysis, CANX and ADAM19 were assigned to the turquoise module. STEM analysis showed progressive downregulation of seven genes, whereas ADAM19 and LAMA3 showed progressively increasing expression patterns. KLF9 and ADAM19 were significantly upregulated across all time points. LAMA3 and CANX were significantly upregulated only at 72 hpi, while 6 genes were significantly downregulated only at 72 hpi. CDH4 showed sustained downregulation throughout infection. CD151, HDAC2 and ACTA1 were significantly downregulated at both 24 and 72 hpi.

### 3.10 Dynamic regulation of DEGs involved in programmed cell death

**Figure 9:**
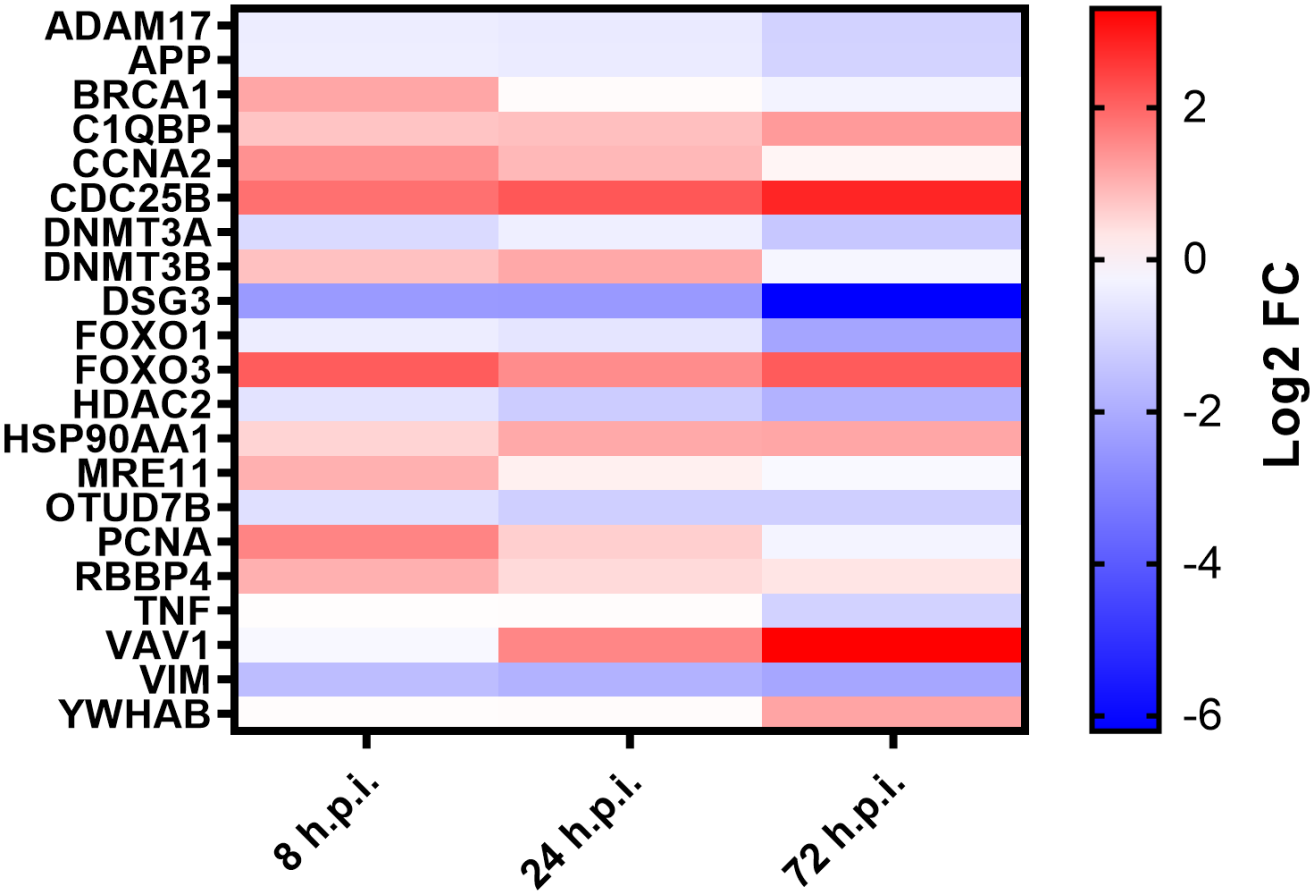
Heat map of the hub genes involved in PCD.

Within the programmed cell death (PCD) pathway, 21 hub genes were identified (**Figure 9**; **Supplementary Table 9**). Five were clustered in the green module and three in the turquoise module. STEM analysis revealed progressive downregulation of 10 genes and progressive upregulation of 3 genes. Five hub genes were significantly upregulated at 8 hpi. CDC25B and FOXO3 showed sustained upregulation, whereas DSG3 and VIM were consistently downregulated throughout infection. Five hub genes were significantly downregulated at 72 hpi. HDAC2 and OTUD7B were significantly downregulated at 24 and 72 hpi. C1QBP and YWHAB were significantly upregulated at 72 hpi, and HSP90AA1 and VAV1 were also significantly upregulated at 24 and 72 hpi.

### 3.11 Dynamic regulation of DEGs involved in PI3K/AKT signaling

**Figure 10:**
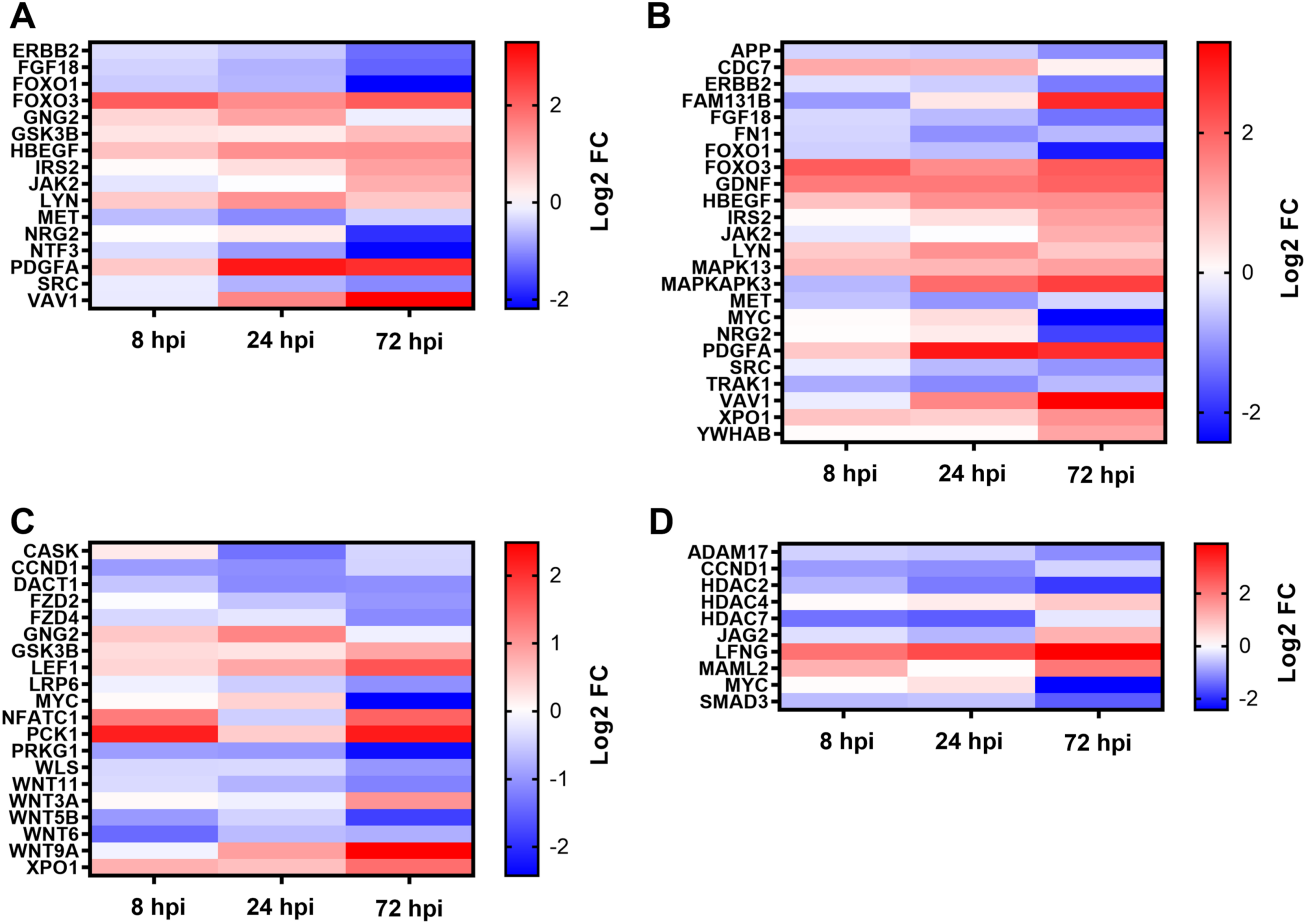
**(A)** Heat map of the hub genes in the PI3K/AKT signaling pathway. **(B)** Heat map of the hub genes in the MAPK signaling pathway. **(C)** Heat map of hub genes in the WNT signaling pathway. **(D)** Heat map of hub genes in the Notch signaling pathway.

The PI3K/AKT pathway regulates cell survival, metabolism, cytoskeleton remodeling, endocytosis, and innate immune responses. A total of 16 hub genes were identified within this pathway (**Figure 10A**; **Supplementary Table 9**). In WGCNA analysis, VAV1, HBEGF and GSK3B were assigned to the turquoise module. STEM analysis revealed progressive downregulation of six genes, whereas VAV1, JAK2 and IRS2 showed increasing expression over the course of infection. FOXO3 showed sustained upregulation at all time points. By 72 hpi, six genes were significantly downregulated, while HBEGF, PDGFA, and VAV1 were significantly upregulated at both 24 and 72 hpi.

### 3.12 Dynamic regulation of DEGs involved in MAPK signaling

The MAPK (Mitogen-Activated Protein Kinase) signaling pathway mediates signal transduction surface receptors to the nucleus and regulates proliferation, differentiation, stress responses, and apoptosis. In the pathway, 24 hub genes were identified (**Figure 10B**; **Supplementary Table 9**). Five genes were clustered in the turquoise module, whereas CDC7 was assigned in the green module. STEM analysis revealed progressive downregulation of 6 genes and progressive upregulation of another 6 genes over the course of infection. FN1, TRAK1, and MET were significantly downregulated at 24 hpi, and 7 hub genes were downregulated at 72 hpi. In contrast, five hub genes were significantly upregulated at 72 hpi. MAPKAPK3, VAV1 and PDGFA were significantly upregulated at 24 and 72 hpi, while CDC7 was significantly upregulated at 8 and 24 hpi. FOXO3 and GDNF showed sustained upregulation throughout infection.

### 3.13 Dynamic regulation of DEGs involved in WNT signaling

The WNT signaling pathway is a conserved cell-to-cell communication system that interacts with NF-κB and interferon pathways to regulate innate immunity and antiviral responses. In fish, Wnt/FZD signaling has also been linked to immune regulation and host–virus interactions during infection. A total of 20 hub genes were identified within the WNT signaling pathway (**Figure 10C**; **Supplementary Table 9**). In the WGCNA analysis, LEF1, XPO1, PCK1, and GSK3B were clustered in the turquoise module. STEM analysis revealed progressive downregulation of PRKG1 and MYC, whereas LEF1 and XPO1 showed increasing expression over time. WNT6 was significantly downregulated at 8 hpi. By 72 hpi, eight genes were significantly downregulated. DACT1 was significantly downregulated at 24 and 72 hpi, whereas CCND1 and CASK were significantly downregulated at 24 hpi. In contrast, WNT3A, XPO1, and LEF1 were significantly upregulated at 72 hpi, and GNG2 was significantly upregulated at 24 hpi. RUNX1 showed sustained upregulation throughout the course of infection.

### 3.14 Dynamic regulation of DEGs involved in Notch signaling

Notch signaling is a conserved cell–cell communication pathway that regulates innate and adaptive immune responses and can influence antiviral defenses or viral persistence. In fish, it has also been linked to immune regulation, tissue repair, and host–virus interactions during infection. Ten hub genes were identified within the Notch signaling pathway (**Figure 10D**; **Supplementary Table 9**). Among these only LFNG was assigned to the turquoise module. STEM analysis showed progressive downregulation of HDAC2, SMAD3, and MYC, whereas HDAC7, JAG2, and LFNG showed increasing expression over time. At 72 hpi, SMAD3, ADAM17, and MYC were significantly downregulated. HDAC2 was significantly downregulated at both 24 and 72 hpi, while HDAC7 was downregulated at 8 and 24 hpi. CCND1 was significantly downregulated at 24 hpi. In contrast, JAG2 was significantly upregulated at 72 hpi, MAML2 at 8 and 72 hpi, and LFNG showed sustained upregulation across all time points.

### 3.15 Dynamic regulation of DEGs involved in PRR signaling pathway

**Figure 11:**
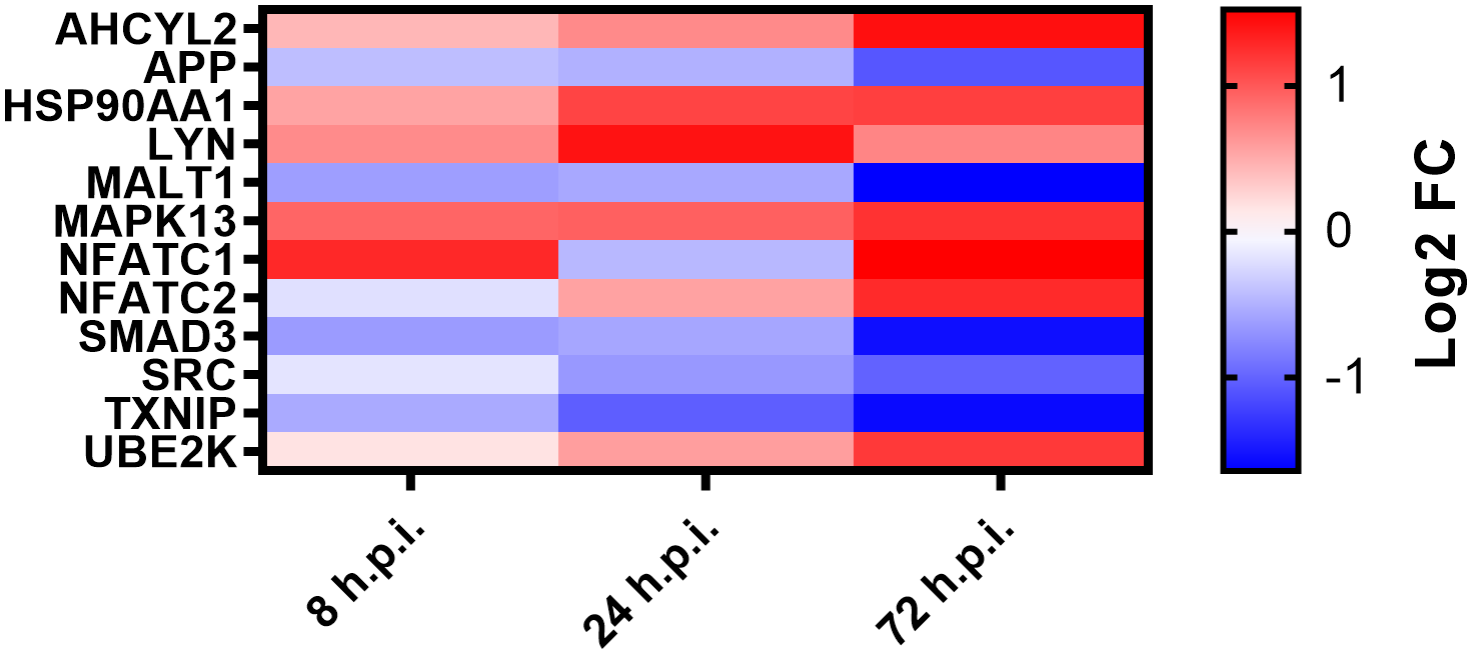
Heat map of the hub genes in the PRR signaling pathway.

The pattern recognition receptor (PRR) signaling pathway is a key component of innate immunity that detects invading pathogens and initiates antiviral responses. PRRs, including Toll–like receptors (TLRs), NOD-like receptors (NLRs), and RIG-I-like receptors (RLRs), recognize pathogen-associated molecular patterns (PAMPs), and trigger downstream signaling cascades. Twelve hub genes were identified within the PRR signaling pathway (**Figure 11**; **Supplementary Table 9**). In the WGCNA analysis, AHCYL2, UBE2K, NFATC2 and MAPK13 were clustered in the turquoise module. STEM analysis revealed progressive downregulation of MALT1, TXNIP and SMAD3, whereas AHCYL2 and UBE2K showed increasing expression over time. At 72 hpi, APP, MALT1, SMAD3, and SRC were significantly downregulated, and TXNIP was reduced at both 24 and 72 hpi. In contrast, MAPK13, AHCYL2, NFATC2, and UBE2K were significantly upregulated at 72 hpi. HSP90AA1 was significantly upregulated at 24 and 72 hpi, while LYN was upregulated at 24 hpi.

### 3.16 Validation of selected DEGs

**Figure 12:**
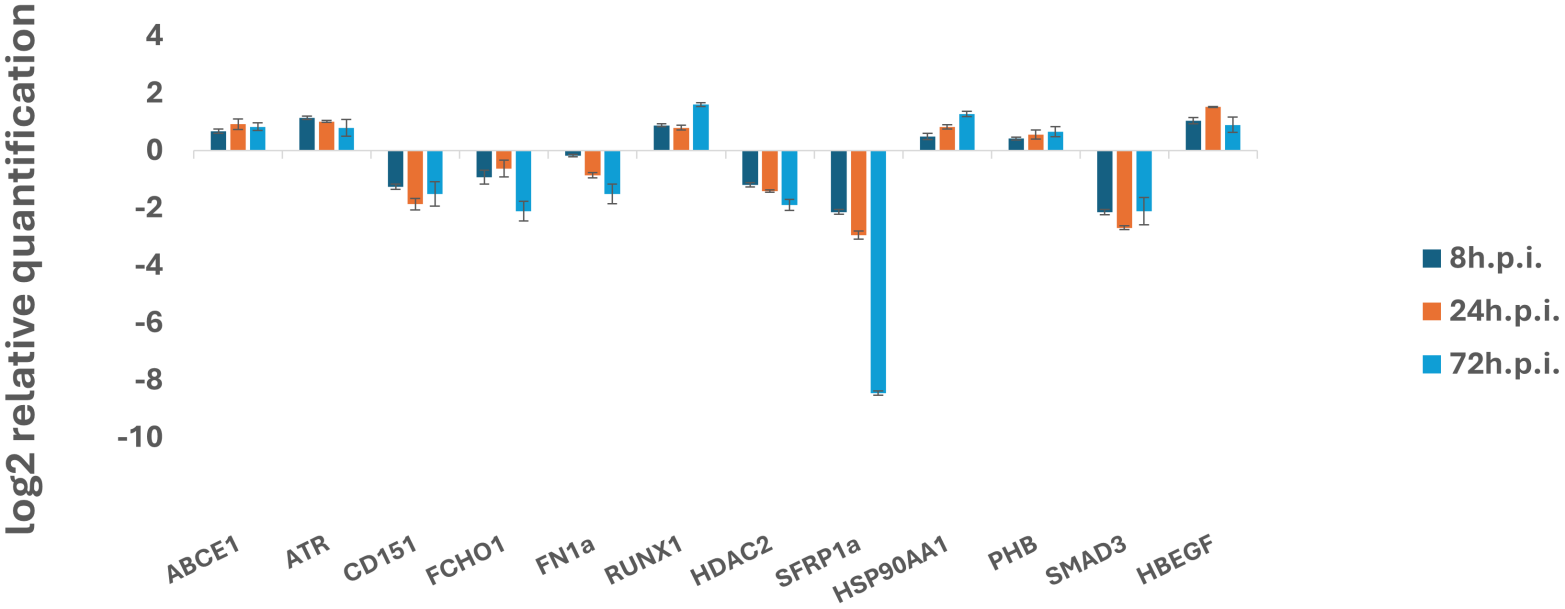
Expression validation of selected DEGs in ASBK-1 cells at 8, 24, and 72 hpi. Bar plots show the log2 fold changes in transcript levels relative to mock controls. Error bars indicate variability among biological replicates. Statistical analysis confirmed that all selected candidate genes exhibited significant differential modulation across all sampled time points compared with mock-infected controls (*p* < 0.05).

To validate the RNA-seq results, qRT-PCR was performed on twelve representative genes (ABCE1, ATR, CD151, FCHO1, FN1a, RUNX1, HDAC2, SFRP1a, HSP90AA1, PHB, SMAD3, and HBEGF) selected from key enrichment pathways. Primer specificity was confirmed by melt curve analysis, which showed single, specific amplification products for each gene (**Figure 12**). Statistical analysis confirmed that all selected candidate genes exhibited significant differential modulation across all sampled time points compared with mock-infected controls (*p* < 0.05). The results showed high correlation between RNA-Seq and RT-qPCR data, thereby validating the accuracy and reliability of the transcriptome assembly and confirming that the identified DEGs reflect the true physiological state of the host during infection.

## 4 Discussion

This study establishes Asian seabass kidney cells as a highly permissive *in vitro* model for host response to SDDV infection. By integrating differential expression analysis, PPI networks, STEM temporal clustering, and WGCNA, we demonstrate that SDDV infection induces coordinated yet heterogeneous remodeling of immune signaling, viral entry pathways, cell–cell communication, programmed cell death, and cell cycle regulation. Together, these findings provide a systems-level view of how SDDV reshapes host pathways to support viral replication while modulating antiviral defenses.

### 4.1 ASBK cells as a reliable model for SDDV infection

Our data confirm that ASBK-1 cells provide a robust and reproducible platform for SDDV propagation and host-response profiling. Further, transcriptomic analysis revealed coordinated modulation of nuclear transport and replication stress pathways, including NUP188, NDC1, XPO1, NPM1, LIG1, together with regulation of key cell-cycle genes such as CDC25B, CCNA2, PCNA. Changes were also observed in pathways associated with viral attachment and entry, particularly extracellular matrix (ECM) components and endocytic regulators. Dynamic regulation of receptor-linked signaling mediators (e.g., SRC, MET downregulation; and stage-specific induction of HBEGF and VAV1) further demonstrates that this model captures signaling–cytoskeletal coupling relevant to viral trafficking. Together, ASBK-1 cells provide a tractable system for mechanistic studies of SDDV pathogenesis.

### 4.2 Dynamic modulation of innate immune signaling during SDDV infection

SDDV infection was associated with an early transcriptional response, including induction of ISG20 (16), POLR3A (17), and IL12B (18), consistent with activation of innate sensing pathways following viral exposure. POLR3A is linked to RNA polymerase III–RIG-I signaling, supporting early engagement of nucleic acid-driven antiviral programs. However, the immune response evolved over time. At 72 hpi, pro-inflammatory mediators including TNF and TXNIP, were reduced, suggesting attenuation or reprogramming of inflammatory signaling. Given the association of TXNIP with inflammasome activity, its late suppression may reflect feedback mechanisms that limit excessive inflammation. In contrast, upstream signaling regulators such as VAV1 and MAPKAPK3 remained elevated at 24–72 hpi, indicating immune recalibration rather than sustained inflammatory activation. Late modulation of WNT (e.g., WNT6, WNT3A, LEF1) and Notch components (e.g., LFNG) further supports pathway remodeling that may influence inflammatory and cellular responsiveness during prolonged infection. In parallel, changes in MAPK and PI3K/AKT pathway hubs support stress-adaptive and survival-associated signaling states rather than uniform antiviral amplification.

A pronounced transcriptional response was observed at 8 hpi despite viral titers being below the detection limit. In our experimental setup, cells were washed after viral adsorption to remove unbound virus and residual inoculum, thereby reducing the contribution of extracellular components. However, as the viral stock was prepared by freeze–thaw lysis without further purification, host-derived debris and damage-associated molecular patterns (DAMPs) may still be present and could contribute to the observed early transcriptional changes. Therefore, while the early induction of immune-related genes likely reflects host sensing of infection, a contribution from DAMP-mediated signaling cannot be fully excluded. The comparable number of DEGs observed at 8 and 24 hpi suggests that the early response represents a rapid sensing phase, likely driven by PRR-mediated signaling and cellular stress responses following viral exposure. In contrast, later time points showed more structured and pathway-specific transcriptional changes associated with active viral replication, indicating a transition from initial sensing to sustained host–virus interaction and cellular reprogramming.

### 4.3 Extracellular matrix remodeling and endocytic pathway rewiring

The transcriptomic signature associated with virus attachment and entry indicates selective remodeling of ECM components, heparan sulfate proteoglycan (HSPG)-associated genes, and clathrin-mediated endocytosis (CME) regulators. Isoform-specific regulation of laminins (LAMA1 and LAMA3 upregulated; LAMA2 downregulated) and differential expression of glypicans (GPC5 induced; GPC1 suppressed) suggest targeted reshaping of cell-surface microdomains that may influence virion docking and receptor clustering.

CME-related components were also differentially regulated (e.g., HIP1 and ITSN2 upregulated; FCHO1 and PICALM downregulated), indicating changes in membrane trafficking and coated-pit dynamics during infection. Given that viral entry relies on pre-existing host machinery, these transcriptional changes are unlikely to directly reflect the initial entry process. Instead, they more likely represent infection-associated remodeling of endocytic pathways, receptor turnover and intracellular trafficking. Persistent suppression of LRP2 supports broader alterations in receptor-mediated uptake (19). In addition, the transition of VAV1 from early suppression to later induction may reflect stage-dependent coupling between signaling pathways and cytoskeletal remodeling during viral trafficking and replication.

### 4.4 Effect of cell-cycle control on SDDV infection

Many viruses manipulate host cell cycle machinery to optimize replication. SDDV infection was associated with coordinated modulation of nuclear pore components (NUP188, NDC1) and the nuclear export factor XPO1, suggesting altered nucleocytoplasmic trafficking (20). Sustained upregulation of NDC1 and NUP188 implies reorganization of nuclear pore complex architecture and increased XPO1 expression suggests enhanced nuclear export capacity, which can influence both viral replication and host transcriptional control (21). Cell cycle regulators exhibited biphasic patterns. Early induction of CDC25B and CCNA2 is consistent with pro-cycle signaling, whereas late reduction of PCNA indicates replication stress and possible checkpoint activation. Sustained NPM1 upregulation further supports persistent cellular stress during infection.

### 4.5 SDDV-driven remodeling of cell–cell communication

Effective cell–cell communication relies on intact adhesion complexes (e.g., cadherins, Ig-CAMs), integrin–ECM interactions, and cytoskeletal anchoring, which together maintain barrier integrity, signal coordination, and immune surveillance. Disruption of these systems during viral infection facilitates viral spread, immune evasion, and tissue damage. During SDDV infection, genes involved in cell–cell communication were predominantly suppressed, including adhesion molecules (CD151, CDH4, CADM2), cytoskeletal regulators (ACTA1, FLNC), and integrin-associated adaptors (FERMT2). This pattern suggests weakening of intracellular junctional integrity during infection. In contrast, induction of KLF9 and late upregulation of CANX suggest activation of stress-responsive transcriptional programs and ER-associated pathways during sustained viral replication.

### 4.6 SDDV-induced programmed cell death

PCD represents a critical intersection between antiviral defense and viral persistence. Recent evidence indicates that SDDV infection can induce ferroptosis (22). Consistent with this, our data revealed regulation of key ferroptosis-associated genes, including ACSL1, ACSL6, GPX4, and SLC7A11. Although these genes did not emerge as central hub genes, they play critical roles in the execution of ferroptosis. The hub genes characterized by metabolic changes (e.g. HK1 induction), activation of stress-associated kinases (e.g. MAPK13, MAPKAPK3), and suppression of structural markers (e.g. VIM, FN1, and DSG3) at later stages are consistent with a potential pro-ferroptotic milieu. Further functional studies are required to validate these observations. In addition, canonical executioner caspases were not prominent among hub genes. Several pro-apoptotic mediators including APP (23), FOXO1 (24) and TNF, were reduced at later time points, whereas genes with reported pro-survival functions (CDC25B (25), FOXO3 (26), HSP90AA1 (27), YWHAB (28)) were maintained or elevated. Overall, these patterns suggest delayed or restrained apoptosis, potentially allowing sustained viral production before terminal cell damage.

In contrast, co-expressed genes within the WGCNA green module, including CDC7, MRE11, BRCA1, PCNA, CCNF, reflect DNA damage and replication stress signatures that may lower the threshold for intrinsic programmed cell death during viral infection.

### 4.7 Study limitations and genomic considerations

While the ASBK-1 cell line provides a robust platform for studying SDDV, several limitations should be considered when interpreting these results. This study is based on an *in vitro* system, which does not fully reflect the complexity of SDDV infection *in vivo*. In fish, the disease involves tissue-specific responses, including skin and scale pathology, as well as broader systemic effects. These aspects are not captured in a single cell model, and therefore the findings should be interpreted with this in mind. Further *in vivo* studies will be needed to assess how these observations relate to disease progression in ASB.

Another important consideration is the genomic background of teleost fish. The teleost-specific whole-genome duplication (Ts3R) has resulted in many paralogous genes in the Asian seabass genome. These genes can retain overlapping functions or evolve new roles, which makes direct comparison with mammalian immune responses less straightforward. In addition, fish possess a diverse and expanded set of Toll-like receptors (TLRs) and interferons (IFNs), many of which lack clear mammalian counterparts. Because our enrichment and protein–protein interaction analyses rely on databases that are largely based on human and mouse data, some fish-specific features may be underrepresented or interpreted with limited resolution.

Finally, although this study provides a broad transcriptional overview of host responses to SDDV infection, functional validation of key candidate genes was beyond the scope of the present work. Future studies using fish-specific functional annotations and gene knockout approaches will be important to determine whether these transcriptional changes reflect conserved vertebrate responses or teleost-specific adaptations to SDDV. The use of improved Asian seabass genome annotations will further strengthen these analyses.

## 5 Conclusion

In summary, SDDV infection in ASBK-1 cells induced an early innate immune response together with remodeling of pathways linked to viral entry. As infection progressed, broader changes emerged, including rewiring of signaling, metabolic, and structural pathways. Late-stage infection was characterized by immune recalibration, altered nuclear transport and cell cycle regulation, replication stress, and selective activation of programmed cell death pathways. These findings provide mechanistic insight into how SDDV coordinates viral replication with host cellular reprogramming and highlight ASBK-1 cells as a valuable platform for studying SDDV pathogenesis and evaluating potential antiviral strategies. However, the Ts3R, together with the presence of fish-specific TLR and IFN repertoires, suggests that some observed transcriptional changes may reflect lineage-specific adaptations rather than conserved vertebrate responses. Future functional studies will be required to clarify the specific roles of individual genes in regulating viral replication and host responses.

## Data Availability

The datasets presented in this study can be found in online repositories. The names of the repository/repositories and accession number(s) can be found in the article/**Supplementary Material**.
