# Supplementary material for: Temporal transcriptomic analysis of scale drop disease virus in Asian seabass kidney cells reveals host immune and signaling changes
Source: Front Immunol. 2026 Jun 9;17:1819983. doi: 10.3389/fimmu.2026.1819983 (PMC13286770; doi:10.3389/fimmu.2026.1819983)
Supplement: Supplementary file 1 [file DataSheet1.docx]

Supplementary Material

# Supplementary Figures


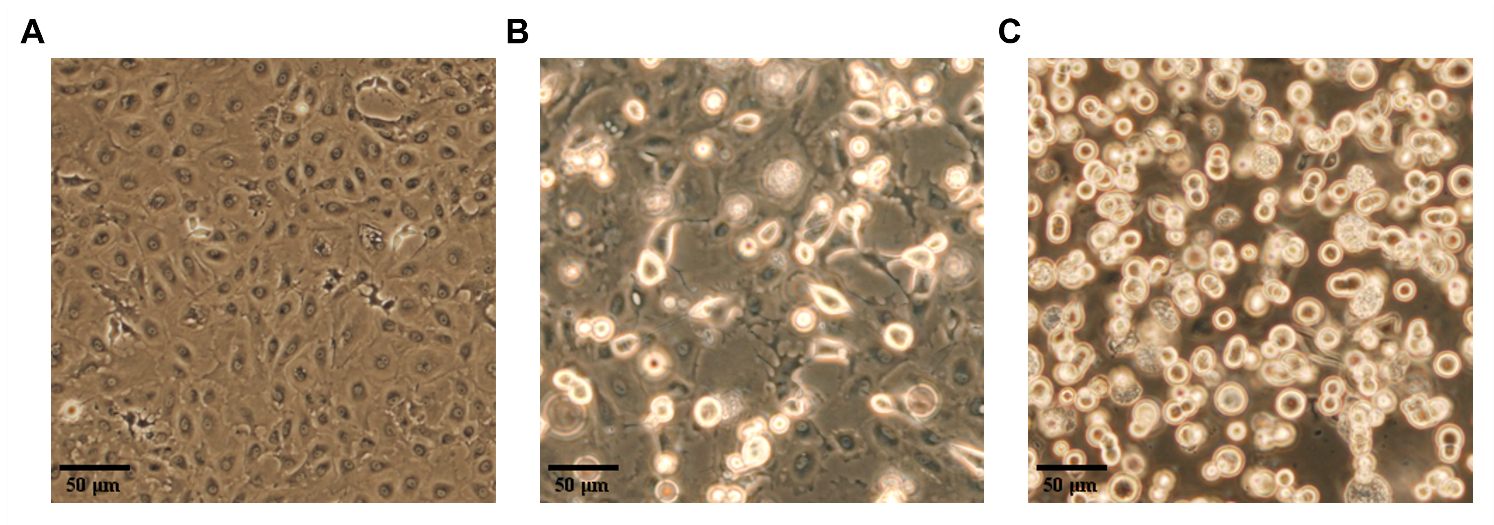


**Figure S1**. ASBK-1 cells infected with SDDV. (**A**) Uninfected ASBK-1 cells (control). (**B**) SDDV -infected ASBK-1 cells showing cytopathic effects on day 2 post-infection at 28 °C. (**C**) SDDV -infected ASBK-1 cells showing extensive cell detachment and monolayers disruption on day 3 post-infection at 28 °C. Scale bar = 50 µm.


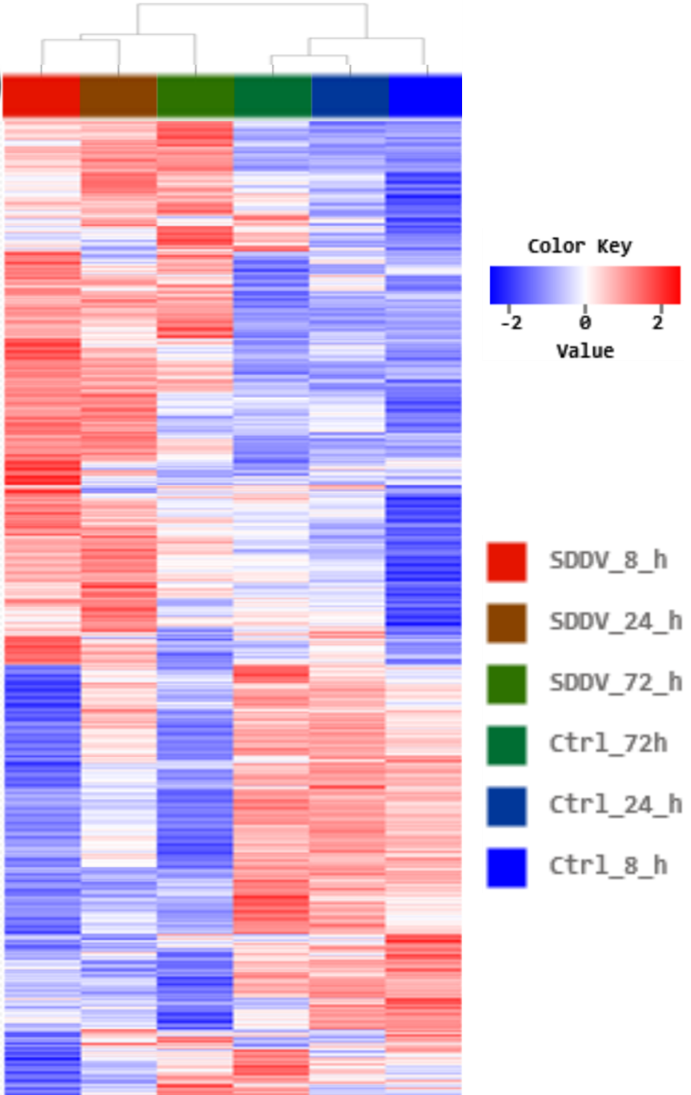


**Figure S2**. Hierarchical clustering heatmap illustrating global transcriptomic changes in mock- and SDDV-infected ASBK-1 cells at 8, 24, and 72 hpi.


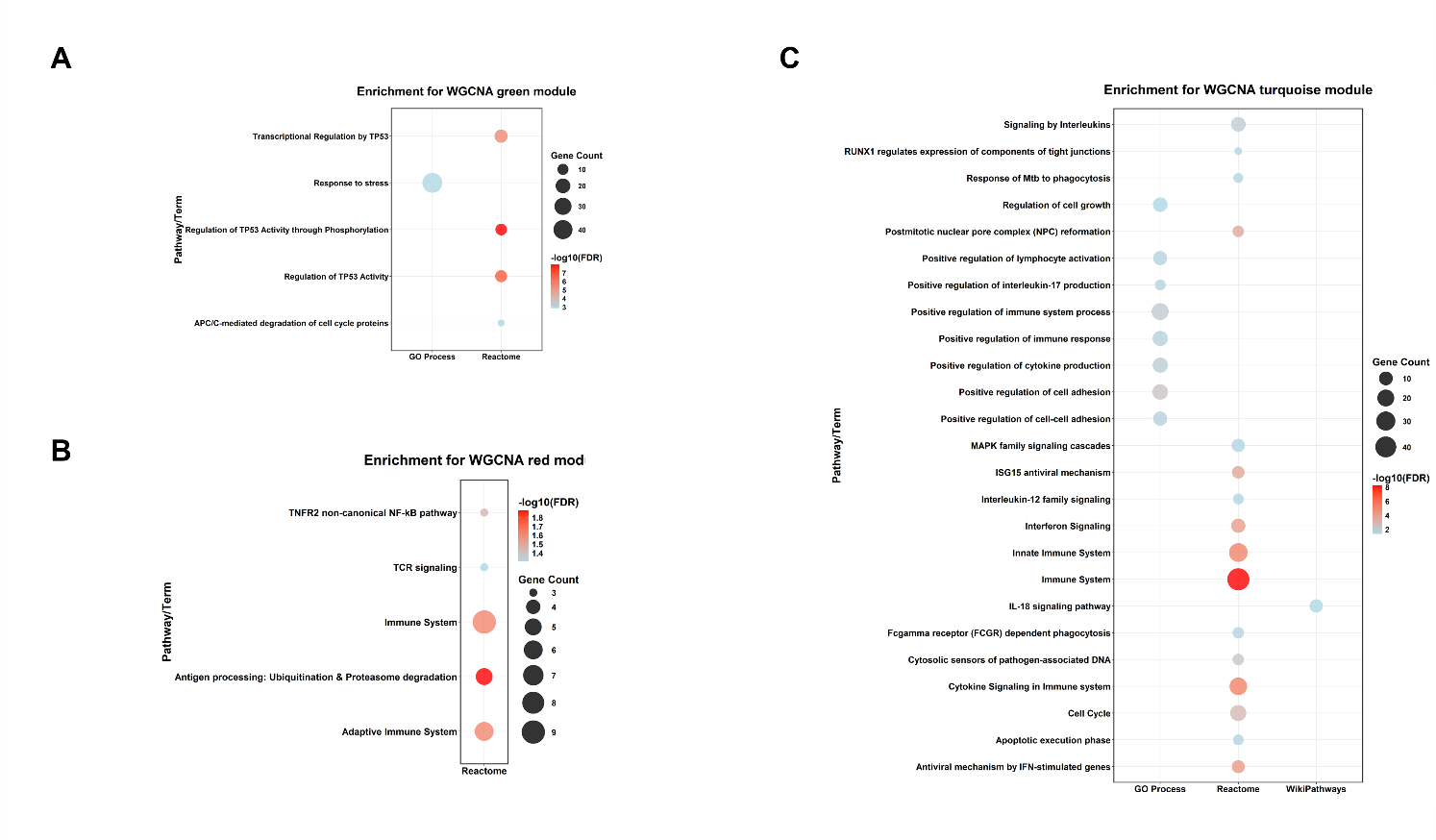


**Figure S3.** Functional enrichment analysis of WGCNA modules. Each bubble corresponds to an enriched biological term. Bubble size reflects the number of genes assigned to that term, while bubble colour represents statistical significance, shown as –log10(FDR). (**A)** Enrichment of green module. (**B)** Enrichment of red module. (**C)** Enrichment of turquoise module.


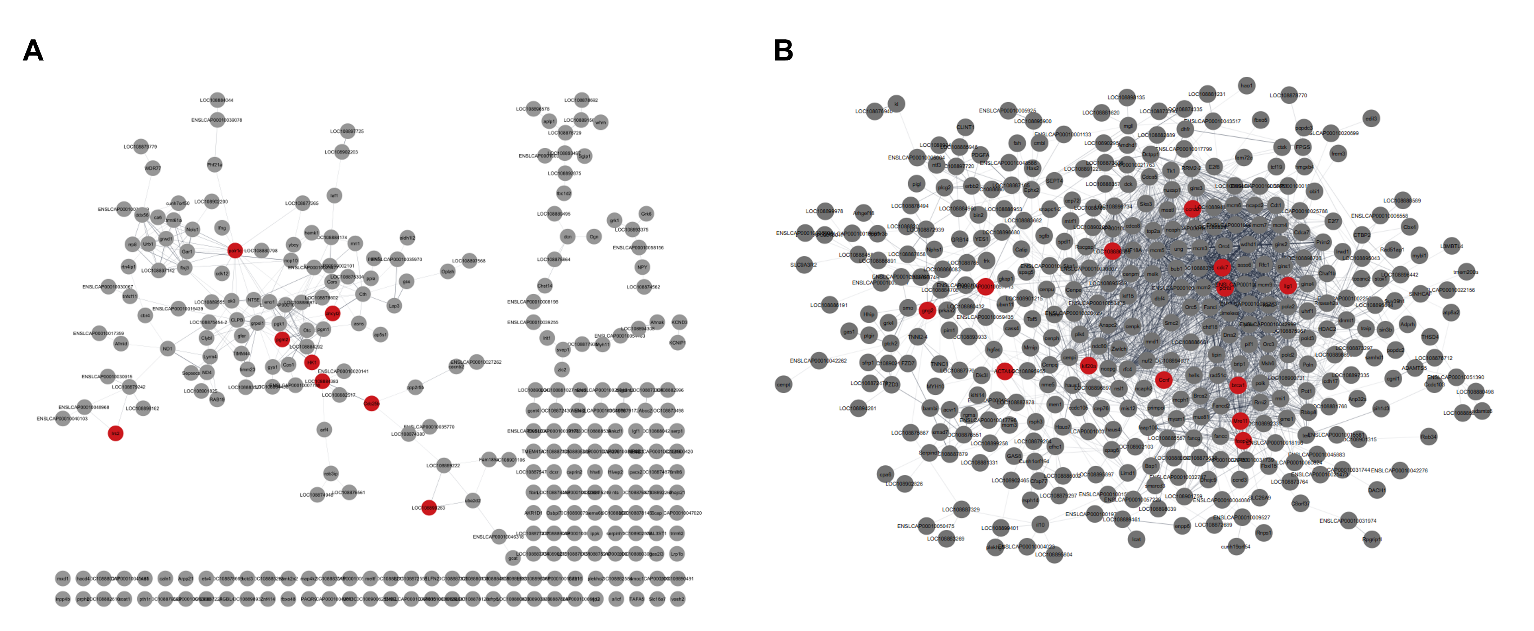


**Figure S4**. StringDB PPI network analysis and hub genes (red nodes) of the STEM analysis genes (see full figures in SVG file). The PPI analysis and hub genes identification are based on Asian sea bass. The hub genes were then mapped to the segregated human pathways. (**A**) Hub genes in STEM uptrend group. (**B**) Hub genes in in STEM downtrend group.


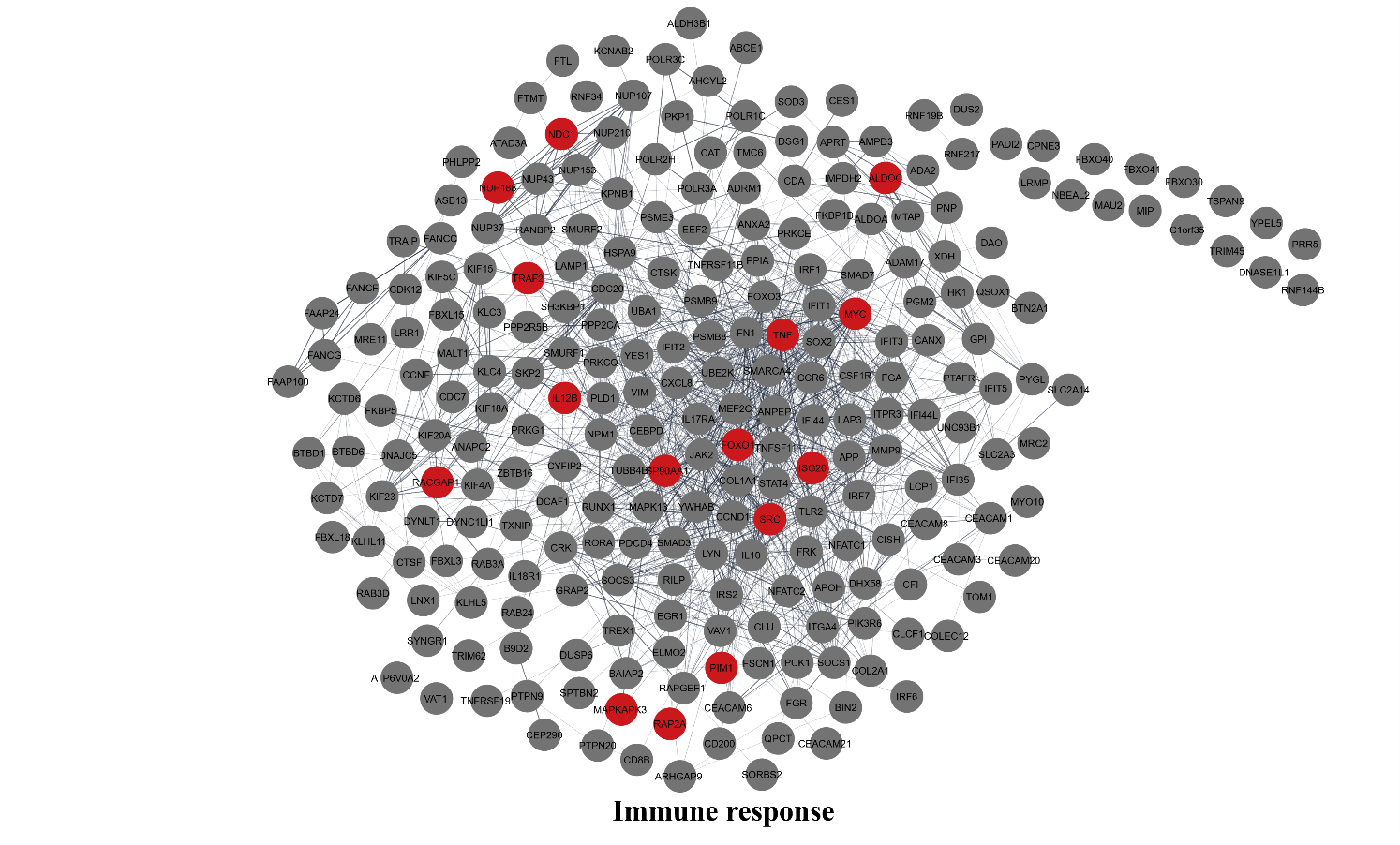


**Figure S5.** StringDB PPI network analysis and hub genes (red nodes) of the segregated genes in immune response.


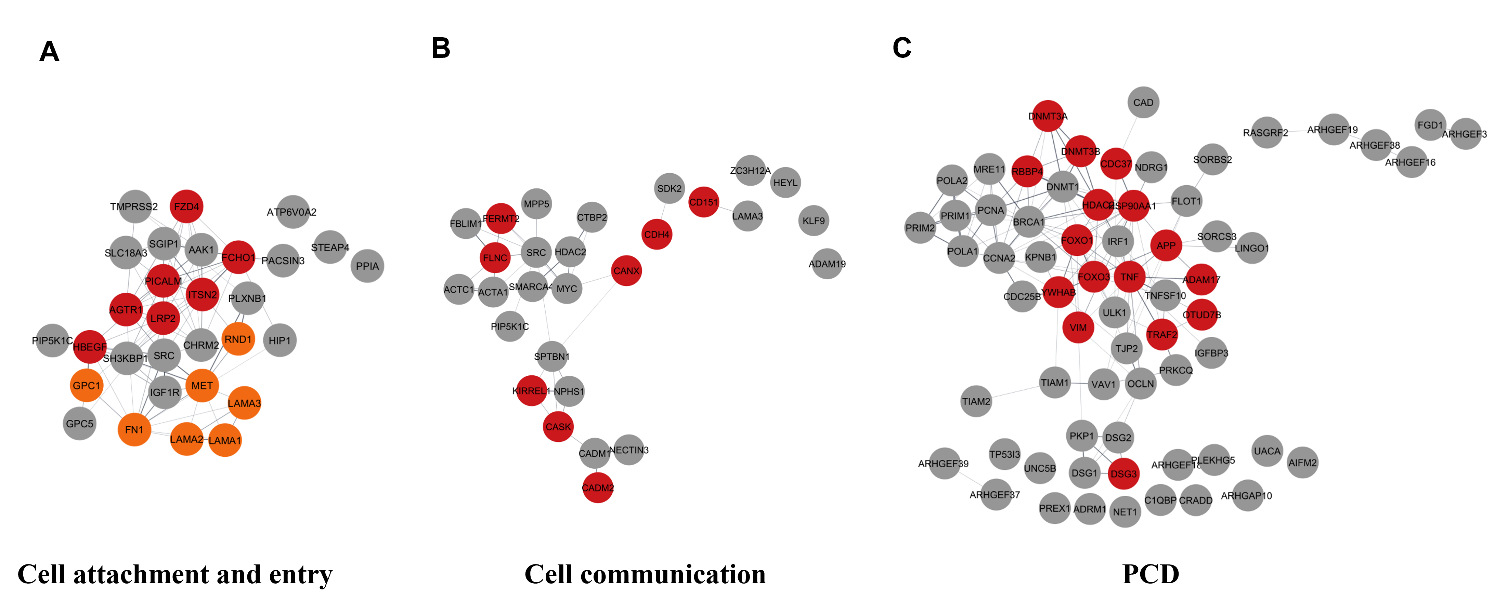


**Figure S6**. StringDB PPI network analysis and hub genes (red and orange nodes) of the segregated genes in (**A**) Cell attachment (orange) and entry (red), (**B**) Cell communication, (**C**) PCD.


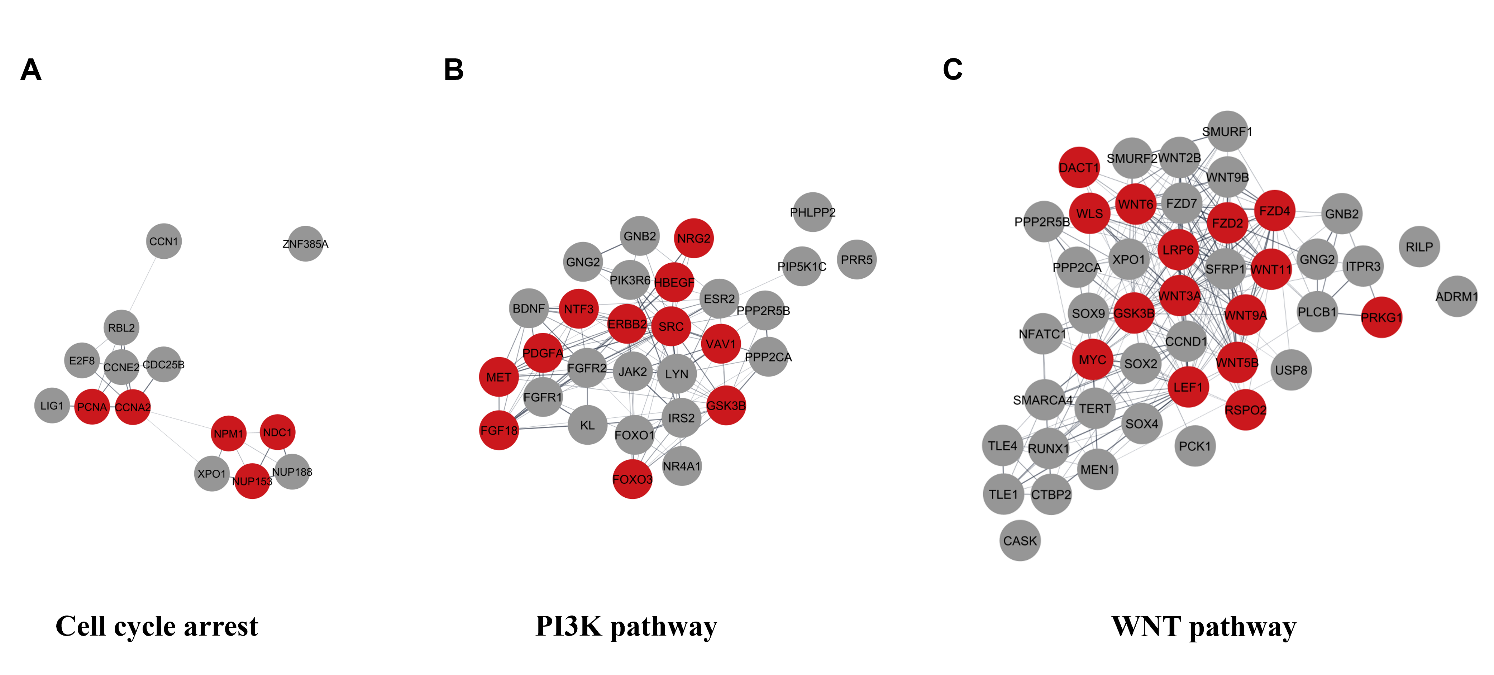


**Figure S7.** StringDB PPI network analysis and hub genes (red nodes) of the segregated genes in (**A**) Cell cycle arrest, (**B**) PI3K pathway and (**C**) WNT pathway.


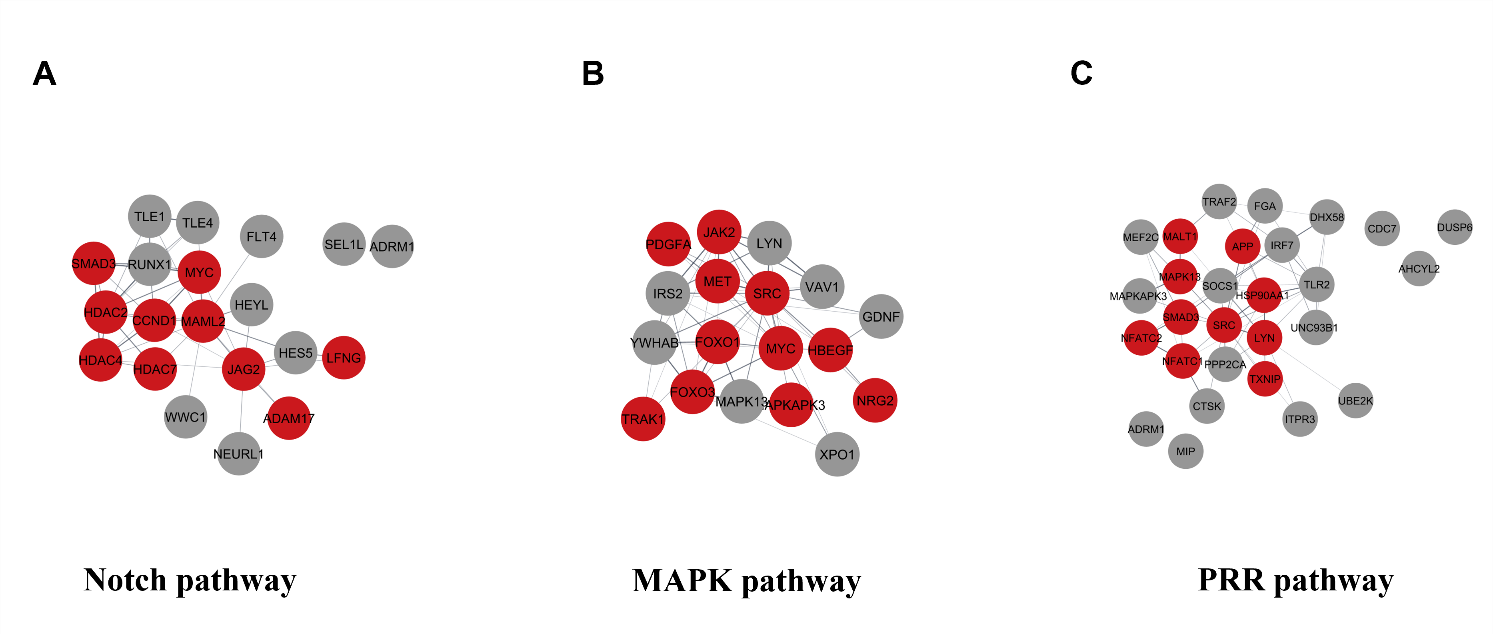


**Figure S8.** StringDB PPI network analysis and hub genes (red nodes) of the segregated genes in (**A**)Notch pathway, (**B**) MAPK pathway and (**C**) PRR pathway.
